# The Effect of Genetic Variation on the Placental Transcriptome in Humans

**DOI:** 10.3389/fgene.2019.00550

**Published:** 2019-06-11

**Authors:** Triin Kikas, Kristiina Rull, Robin N. Beaumont, Rachel M. Freathy, Maris Laan

**Affiliations:** ^1^Human Genetics Research Group, Institute of Biomedicine and Translational Medicine, University of Tartu, Tartu, Estonia; ^2^Women’s Clinic, Tartu University Hospital, Tartu, Estonia; ^3^Department of Obstetrics and Gynecology, University of Tartu, Tartu, Estonia; ^4^Institute of Biomedical and Clinical Science, University of Exeter Medical School, University of Exeter, Exeter, United Kingdom

**Keywords:** human placenta, *cis*-eQTL, complex traits, REPROMETA, ALSPAC, HAPPY PREGNANCY

## Abstract

The knowledge of genetic variants shaping human placental transcriptome is limited and they are not cataloged in the Genotype-Tissue Expression project. So far, only one whole genome analysis of placental expression quantitative trait loci (eQTLs) has been published by Peng et al. (2017) with no external independent validation. We report the second study on the landscape of placental eQTLs. The study aimed to generate a high-confidence list of placental *cis*-eQTLs and to investigate their potential functional implications. Analysis of *cis*-eQTLs (±100 kbp from the gene) utilized 40 placental RNA sequencing and respective whole genome genotyping datasets. The identified 199 placental *cis*-eSNPs represented 88 independent eQTL signals (FDR < 5%). The most significant placental eQTLs (FDR < 10^-5^) modulated the expression of ribosomal protein RPL9, transcription factor ZSCAN9 and aminopeptidase ERAP2. The analysis confirmed 50 eSNP-eGenes pairs reported by Peng et al. (2017) and thus, can be claimed as robust placental eQTL signals. The study identified also 13 novel placental eGenes. Among these, *ZSCAN9* is modulated by several eSNPs (experimentally validated: rs1150707) that have been also shown to affect the methylation level of genes variably escaping X-chromosomal inactivation. The identified 63 placental eGenes exhibited mostly mixed or ubiquitous expression. Functional enrichment analysis highlighted 35 Gene Ontology categories with the top ranking pathways “ruffle membrane” (FDR = 1.81 × 10^-2^) contributing to the formation of motile cell surface and “ATPase activity, coupled” (FDR = 2.88 × 10^-2^), critical for the membrane transport. Placental eGenes were also significantly enriched in pathways implicated in development, signaling and immune function. However, this study was not able to confirm a significant overrepresentation of genome-wide association studies top hits among the placental eSNP and eGenes, reported by Peng et al. (2017). The identified eSNPs were further analyzed in association with newborn and pregnancy traits. In the discovery step, a suggestive association was detected between the eQTL of *ALPG* (rs11678251) and reduced placental, newborn’s and infant’s weight. Meta-analysis across REPROMETA, HAPPY PREGNANCY, ALSPAC cohorts (*n* = 6830) did not replicate these findings. In summary, the study emphasizes the role of genetic variation in driving the transcriptome profile of the human placenta and the importance to explore further its functional implications.

## Introduction

The placenta is a unique mammal-specific organ. It serves the developing fetus during its short intrauterine period not only as a mediator of maternal resources, but also as a contributor to its developmental programming, growth, and maturation for the postnatal life. Throughout its restricted lifetime, coordinated dynamics of placental gene expression across the entire gestation reflects the growing needs of the fetus in order to guarantee the normal course of pregnancy (Winn et al., 2007; Uusküla et al., 2012). Disturbances in placental gene expression at any gestational time point may lead to or reflect placental malfunction and various pregnancy-related complications of the mother and/or the fetus, such as recurrent pregnancy loss, preeclampsia, intra-uterine growth restriction (Sõber et al., 2015, 2016).

Several modulators of placental gene expression have been investigated in order to uncover their role in placental function and in predisposition to develop pregnancy complications. Amongst the most studied effectors are disturbances in intrauterine conditions [e.g., hypoxia (Soares et al., 2017)], communication at the maternal-fetal interface (Pavličev et al., 2017), placental microRNAs (Deshpande and Balasinor, 2018) and epigenetic alterations modulating the transcriptional activity of critical placental genes (Deshpande and Balasinor, 2018). Additional intrinsic genomic factors determining the expression level of one or many genes are expression quantitative trait loci (eQTLs), alternatively referred as eSNPs. Based on the analysis of 48 different human tissues in the framework of the Genotype-Tissue Expression (GTEx) project – the largest coordinated activity aiming to uncover the relationship between genetic variation and gene expression (Lonsdale et al., 2013; Ardlie et al., 2015), the number of eSNPs across the human genome has been estimated to exceed 150,000 (Aguet et al., 2017). The contribution of eSNPs to human pathological conditions has been shown directly in numerous targeted studies [e.g., breast (Li et al., 2013) and pancreatic cancer (Zhang et al., 2018), nephrotic syndrome (Gillies et al., 2018)] and indirectly via enrichment of eSNPs among top associated loci in GWA studies of various diseases and traits, such as psoriasis (Ding et al., 2010) and height (Yengo et al., 2018). As the GTEx project is based on the analysis of donated post-mortem tissues, it does not contain any placental samples. However, it is well acknowledged that eSNPs vary across different tissues (Grundberg et al., 2012; McKenzie et al., 2014) and even as much as 12–40% of them have been estimated to be tissue-specific (Gillies et al., 2018; Zhang et al., 2018). Therefore, it is critical to uncover placental eSNPs in order to understand their role in modulating placental function and risk to gestational disturbances.

Until recently, studies of human placental eSNPs have been restricted to reports focusing on variants modulating transcript levels of single placental genes and their effect on pregnancy outcomes. Of the few studies on placental eQTLs, one has linked variants in *FKBP5* with infant neurobehavioral phenotypes (Paquette et al., 2014), and another reported an association between *STC1* eSNPs and preeclampsia (Juhanson et al., 2016). The only published large-scale placental eQTL study demonstrated that the majority of placental eSNPs are located in the vicinity of the modulated genes (eGenes) (Peng et al., 2017). Analysis of 159 placental transcriptomes identified 3218 (98.9%) eSNPs with *cis*-, but only 35 (1.1%) variants with significant *trans*-effects on the expression of affected genes. The study also demonstrated that >5% of all unique loci associated with any disease in genome-wide association studies (GWAS) represented placental eSNPs. The potential role of placental transcriptome profile in “(mis)programming” the offspring’s risks to human late-onset common diseases was previously discussed in the context of a substantial overlap between GWAS top loci and genes exhibiting high mid-gestational placental expression (Uusküla et al., 2012).

The current study aimed to generate a high-confidence list of genetic variants modulating placental gene expression. The analysis confirmed 50 eSNP-eGenes pairs reported recently by Peng et al. (2017) and identified 13 novel placental eGenes. The variants were further explored for their potential link to complex diseases and newborn growth parameters.

## Materials and Methods

### Ethics Statement

The REPROgrammed fetal and/or maternal METAbolism (REPROMETA) and HAPPY PREGNANCY (full study name “Development of novel non-invasive biomarkers for fertility and healthy pregnancy”) studies were approved by the Ethics Review Committee of Human Research of the University of Tartu, Estonia (Permissions No 146/18, 27.02.2006; 150/33, 18.06.2006; 158/80, 26.03.2007; 221/T-6, 17.12.2012; 286/M-18, 15.10.2018). All study participants were recruited, and the study material was collected at the Women’s Clinic of Tartu University Hospital, Estonia in 2006–2011 (REPROMETA) and in 2013–2015 (HAPPY PREGNANCY). All participants in the REPROMETA and HAPPY PREGNANCY studies were of white European ancestry and living in Estonia. A written informed consent to participate in the study was obtained from each individual prior to recruitment.

Ethical approval for the Avon Longitudinal Study of Parents and Children (ALSPAC) was obtained from the ALSPAC Ethics and Law Committee and the Local Research Ethics Committees. The study recruited pregnant women in Bristol area, United Kingdom in 1991–1992.^1^ Consent for biological samples was collected in accordance with the Human Tissue Act (2004). Informed consent for the use of data collected via questionnaires and clinics was obtained from participants following the recommendations of the ALSPAC Ethics and Law Committee.

All procedures and methods in the three studies have been carried out in compliance with the guidelines of the Declaration of Helsinki.

### REPROMETA Dataset for the Discovery Placental eQTL Analysis

The REPROMETA study represents family trios (mother, father, placenta) recruited before or shortly after delivery of a singleton newborn at the Women’s Clinic of Tartu University Hospital, Estonia. The study was designed to include well-defined, clinically diagnosed diverse scenarios of pregnancy outcomes at term (gestational age 36–42 weeks). The full REPROMETA placental sample set analyzed in the current study (*n* = 336) is comprised of five clinical subgroups: delivery of a small-for-gestational-*age* [SGA, birth weight <10th centile (Sildver et al., 2015); *n* = 65] or large-for-gestational-age newborn (LGA, >90th centile; *n* = 83), cases of maternal gestational diabetes (GD; *n* = 41) or severe late-onset preeclampsia (PE; *n* = 43), and healthy term pregnancies (birth weight 10th–90th centile; *n* = 104). Criteria for the clinical subgrouping are detailed in Supplementary Methods. Clinical and epidemiologic data of the mother and the newborn were collected from medical records.

The current eQTL discovery study utilized previously published RNA sequencing (RNA-Seq) (Sõber et al., 2015; Reiman et al., 2017), and corresponding genome-wide genotyping datasets of 40 term placental samples (Kasak et al., 2015), where each REPROMETA subgroup (NORM, PE, GD, SGA, LGA) was represented by eight placental transcriptomes maximally matched for the gestational age, delivery mode and proportions of male/female newborns (Table 1). Placental sampling, RNA extraction, library sequencing and basic informatics are detailed in the Supplementary Methods and in Sõber et al. (2015) and Reiman et al. (2017).

**Table 1:** Maternal and newborn data of the term placentas utilized *cis*-eQTL discovery analysis (*n* = 40).

Parameter	Unit	eQTL analysis samples
Maternal age	year	28.5 [18-39]
Gestational age at delivery	day	274.0 [260-284]
Newborn birth weight	gram	3587 [2004-4986]
Newborn length	cm	50.2 [45-55]
Placental weight	gram	584.0 [200-1060]
Newborn sex: female/male	*n*	21/19
Delivery mode: EmCS/ECS/Vag	*n* [%]	9/12/19 [22.5%/30.0%/47.5%]
Labor activity: no/yes/NA	*n* [%]	19/20/1 [47.5%/50.0%/2.5%]
Pregnancy complications	*n*	32 [80%] PE, GDM, SGA, LGA: 8 each


*All cases were recruited, clinical data and placental tissue was collected in the framework of the REPROMETA study at the Women’s Clinic of Tartu University Hospital, Estonia. Detailed information on this sample set and respective the placental RNA-Seq data is provided in Supplementary Methods and original references (Sõber et al., 2015; Reiman et al., 2017). Data is given as mean [minimum–maximum] unless indicated differently. ECS, elective cesarean section; EmCS, emergency cesarean section; GD, gestational diabetes; LGA, large-for-gestational age newborn; NA, not available; PE, preeclampsia; SGA, small-for-gestational age newborn; Vag, vaginal delivery.*

Genome-wide genotyping of the same placental samples was carried out on Illumina HumanOmniExpress-12-v1 BeadChip (>733,000 SNPs, median spacing 2.1 kb; Kasak et al., 2015). Samples were genotyped with an average overall call rate >99% per individual per genotype.

We excluded SNPs if they deviated from Hardy-Weinberg Equilibrium (HWE; *P* < 1 × 10^-6^) or had no minor allele carriers in our dataset. In total of 661,354 genotyped SNPs were taken forward to the next step.

### Bioinformatics for eQTL Detection

The initial unfiltered RNA-Seq dataset of 40 placental samples (Sõber et al., 2015; Reiman et al., 2017) included gene expression data for 53,893 genes (Ensembl v67^2^). Gene expression was quantified using htseq-count (as raw read counts) and then normalized for read depth of the sample. Genes with low expression (applied empirical cutoff: median expression <100 normalized read counts) were excluded. After further filtering out mitochondrial genes, 11,733 genes were retained for the eQTL analysis (Supplementary Figure S1).

As a very low number of placental *trans*-eQTLs was expected (Peng et al., 2017) and the chance to detect spurious *trans*-eQTL associations is high (Yao et al., 2017), our study design focused on *cis*-eQTL discovery to maximize true-positive, functionally relevant findings. In the current study, we defined *cis*-SNPs to locate within ±100 kbp from the gene start/end coordinates as these regions have high probability of containing significant and functionally relevant eQTL hits (Veyrieras et al., 2008). SNP and gene coordinates were extracted from the Ensembl database using BioMart (Ensembl v54) and *cis*-eQTL testing was implemented in Matrix eQTL package for R (Shabalin, 2012). The final number of SNP included into eQTL testing was 353,599 and the total number of performed tests was 659,826 (Supplementary Data S1). Association between gene expression level (quantified as normalized read counts) and *cis*-SNPs was carried out using linear regression adjusted by the pregnancy outcome (normal term or PE, GD, SGA, LGA pregnancy), labor activity and newborn sex. Nominal *P*-values were corrected for multiple testing using a built-in Benjamini and Hochberg FDR method in Matrix eQTL. A statistically significant eSNP-eGene association was defined as FDR < 0.05. For each identified eQTL, the proportion of gene expression variability explained by the SNP was calculated (*R*^2^).

eSNPs were clustered into high LD-groups (*r*^2^ > 0.8) and only the empirically selected lead SNP with the lowest *P*-value was taken forward to represent the LD-group in subsequent analyses. For determining the top eGenes, the lowest *P*-value of any associated eSNP was considered as the *P*-value for the gene.

eGenes were analyzed for the functional enrichment among Gene Ontology pathways (biological processes, cellular components, molecular functions). The statistical significance testing of the enrichment was implemented in FUMA^3^ (Watanabe et al., 2017) and FDR was estimated using Benjamini–Hochberg correction for multiple testing. GWAS data was downloaded from GWAS Catalog.^4^ SNPnexus (Dayem Ullah et al., 2018) was used to determine the closest gene for each GWAS hit. χ^2^ test was used to determine enrichment of eGenes and eSNPs among GWAS results.

### Taqman RT-qPCR Validation of *cis*-eQTLs in REPROMETA Placental Samples

Experimental validation of the discovery study targeted protein coding genes with multiple identified *cis*-SNPs that exhibited low FDR and at least two-fold gene expression difference between the placentas with heterozygote and major homozygote genotypes. The *cis*-eSNPs selected for the validation were rs1150707 (*ZSCAN9* c.568+1990 C > T); rs10044354 (*ERAP2* g.96984791 C > T) and rs11678251 (*ALPG* c.-318 G > A). For these SNPs, an extended REPROMETA placental sample set (*n* = 336; Table 2) was genotyped using Sequenom iPLEX Gold genotyping system (Sequenom, Agena, United States). Primers are provided in Supplementary Table S1. Based on the genotyping outcome, 24 placentas per each eSNP were selected for the Taqman RT-qPCR gene expression quantification (Supplementary Table S2). Taqman RT-qPCR and testing of eQTL effects targeted also three neighboring genes (*ALPP, ERAP1, LNPEP*).

**Table 2:** REPROMETA, HAPPY PREGNANCY, and ALSPAC datasets utilized in the genetic association testing with newborn traits.

Parameter	REPROMETA (*n* = 336)	HAPPY PREGNANCY (*n* = 408)	ALSPAC (*n* = 7669)
Gestational age at delivery	273.4 [196-299] days	271.9 [167-294] days	39.5 [25-47] weeks
Newborn sex (F/M)	170/166	204/204	3732/3937
Placental weight (g)	585.1 [190-1122]	539.4 [190-1132]	657.1 [240-1260]^a^
Birth weight (g)	3545 [990-5740]	3127.1 [560-4418]	3448.8 [645-5640]
Birth length (cm)	49.9 [35-57]	49.0 [29.5-56]	50.8 [34-62]
Head circumference (cm)	34.8 [26-40]	34.2 [22-38]	34.9 [20.3-54]
Chest circumference (cm)	34 [21-43]	32.8 [22.5-37]	NA
Preeclampsia (*n* [%])	43 [12.8%]	8 [2.0%]	150 [2.0%]
Gestational diabetes (*n* [%])	41 [12.2%]	10 [2.5%]	37 [0.5%]
SGA newborn (*n* [%])^b^	65 [19.3%]	158 [38.7%]	771 [10.1%]
LGA newborn (*n* [%])	83 [24.7%]	4 [1.0%]	764 [10.0%]
Maternal age (years)	28.7 [16-43]	28.9 [15.8-47.2]	28.6 [15-44]
Maternal height (cm)	167.1 [150-185]	166.4 [150-186]	164.2 [143.5-188.3]^a^
Maternal pre-pregnancy weight (kg)	67.9 [43-142]	63.3 [40-106]	60.9 [31.6-141.6]
Gestational weight gain (kg)	15.5 [(-3)-40]	12.1 [1.2-28]	12.6 [-6.7 to 37.8]
Maternal smoking: no/yes (*n* [%])	306/21 [91.1%/6.3%]	385/23 [94.4%/5.6%]	5845/1595 [78.6%/21.4%]


*Data is given as mean [minimum–maximum] unless indicated differently. REPROMETA clinical cases were recruited in 2006–2011 and HAPPY PREGNANCY cohort cases in 2013–2015 at the Women’s Clinic of Tartu University Hospital, Estonia. ALSPAC cohort cases were recruited in 1991–1992 in Bristol area, United Kingdom. Detailed information on each study is provided in Materials and Methods. LGA, large-for-gestational age newborn; NA, not available; SGA, small-for-gestational age newborn. ^*a*^Data were available for fewer than half of the samples. ^*b*^SGA includes preeclampsia and preterm cases.*

eQTL testing was conducted as previously, except for using gestational age as an additional cofactor to account for the wider range of gestational age in validation samples. The expression levels for all samples were transformed to represent the fold-change from the median expression of major homozygotes used as the reference. Details for Taqman RT-qPCR validation are provided in Supplementary Methods and Supplementary Table S3.

### HAPPY PREGNANCY and ALSPAC Pregnancy Cohorts and Data

The HAPPY PREGNANCY study recruited prospectively 2334 pregnant women during their first antenatal visit to Women’s Clinic of Tartu University Hospital, Estonia. Longitudinal clinical and epidemiological data includes reproductive history, parental lifestyle, the course and outcome of pregnancy. The current study analyzed 408 placental samples with a specific focus on SGA newborns (*n* = 158; Table 2).

The ALSPAC initially recruited 14,541 pregnant women resident in Avon, United Kingdom, with expected dates of delivery April 1, 1991 to December 31, 1992 (Boyd et al., 2013; Fraser et al., 2013). For all recruited cases medical data from obstetric and perinatal records were documented. From the initial pregnancies, 14,062 resulted in live births. Gestational age at the delivery was recorded the nearest gestational week. The current study analyzed 7669 newborns with available genotype data (Table 2). Please note that the ALSPAC study website contains details of all the data that are available through a fully searchable data dictionary and variable search tool: http://www.bristol.ac.uk/alspac/researchers/our-data/.

The HAPPY PREGNANCY placental samples were genotyped for the rs11678251 (*ALPG* c.-318 G > A) using a pre-made Taqman assay (ID C__27838320_10, Applied Biosystems, Foster City, CA, United States). For the ALSPAC cohort, genotypes of the proxy SNP rs744873 (*r*^2^ = 1.0 with rs11678251) were obtained from the genome-wide array dataset (Boyd et al., 2013).

### Genetic Association Testing

Genetic association analysis was implemented in PLINK, ver. 1.09 (Purcell et al., 2007).^5^ Differences in allelic distributions between term cases of normal pregnancy and gestational complications (PE, GD, SGA, LGA) in the discovery REPROMETA sample set was assessed using logistic regression (additive genetic model).

Genetic association tests with newborn parameters applied linear regression analysis. The REPROMETA placental genotypes (*n* = 336) for three *cis*-eQTLs rs1150707 (*ZSCAN9* c.568+1990 C > T), rs10044354 (*ERAP2* g.96984791 C > T) and rs11678251 (*ALPG*, c.-318 G > A) were tested in association with birth weight and length, placental weight, newborns’ head and chest circumference and postnatal growth (Supplementary Table S4). The tests with the birth parameters were implemented under additive and recessive models, adjusted to newborn sex and gestational age (in days). Based on the outcome of the association testing with birth parameters, only the sex-adjusted recessive model was applied in further association analysis with infant postnatal growth. Infant height and weight had been documented at 6 (*n* = 233) and 12 (*n* = 216) months of age (Supplementary Table S5). For prematurely born children (<259 g days) postnatal growth was adjusted to the gestational age at birth (Supplementary Methods).

Suggestive association of the *ALPG* c.-318 G > A (rs11678251) with the newborn growth parameters in the REPROMETA study (under recessive model) was further assessed in independent cohorts HAPPY PREGNANCY (*n* = 408; Estonia) and ALSPAC (*n* = 7669; United Kingdom). For ALSPAC a proxy SNP rs744873 was analyzed. The test results of individual REPROMETA, HAPPY PREGNANCY and ALSPAC studies were combined in a meta-analysis under fixed effects model.

The obtained nominal *P*-values < 0.05 were considered as suggestive association. The Bonferroni corrected statistical significance level was estimated *P* < 1.2 × 10^-3^ for the 42 tests in the initial REPROMETA study and *P* < 3.6 × 10^-3^ for the 14 tests in the meta-analysis, respectively.

## Results

### Discovery Analysis of Placental Expression Quantitative Trait Loci

**FIGURE 1:**
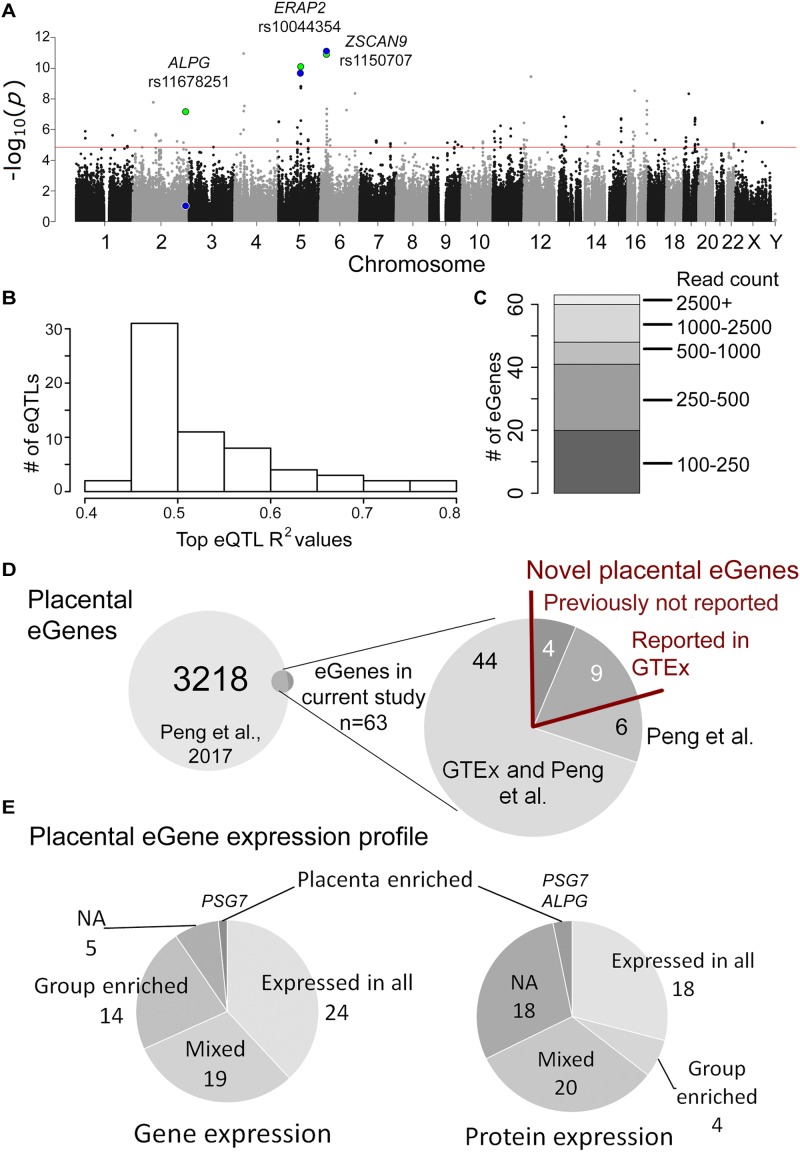
Overview of the placental *cis*-eQTL analysis outcome. **(A)** Manhattan plot representing the landscape of *P*-values from the discovery analysis. The association *P*-values for the three eSNPs-eGene pairs selected for validation experiments are shown for the discovery (green) and validation (blue) analysis. The red line indicates the statistical significance threshold (FDR 5%). **(B)** The proportion of eGene expression (*R*^2^) explained by the eSNPs detected in this study. **(C)** The expression levels of identified placental eGenes, shown in read counts from the RNA-Seq dataset (Sõber et al., 2015). **(D)** The overlap of identified placental eGenes between the current study and Peng et al. (2017). **(E)** Profile of mRNA and protein expression of the placental eGenes according to Human Protein Atlas (www.proteinatlas.org).

Discovery analysis of the placental eQTLs was implemented using published RNA sequencing (Sõber et al., 2015) and whole genome genotyping (Kasak et al., 2015) datasets representing 40 unrelated term placentas collected during the REPROMETA study and representing a diverse range of pregnancy outcomes (Table 1). The analysis design targeted *cis*-eQTLs (±100 kbp from gene) and included genes with sufficient transcript levels (>100 reads; *n* = 11,733 genes after filtering) in order to avoid spurious associations (Supplementary Figure S1). Linear regression testing with 353,599 SNPs (Illumina HumanOmniExpress array) identified 199 placental *cis*-eSNPs corresponding to the applied stringent statistical significance threshold (FDR < 5%) (Figure 1A, Supplementary Table S6A, and Supplementary Data S1). The proportion of gene expression explained by the identified eQTLs (*R*^2^) varied from 0.45 to 0.77 (mean *R*^2^ = 0.52) (Figure 1B). When taking into account linkage disequilibrium (LD; *r*^2^ > 0.8) between the eSNPs, there were in total 88 independent *cis*-eQTL association signals representing 47 singleton eSNPs and 41 eSNP-clusters localized across the genome, except for some smaller chromosomes (#18, 20, 21, Y; Supplementary Figure S2). The densest coverage of independent eSNPs-eGene pairs was detected on chromosome 19 (*n* = 10 SNPs, 0.16 SNP/Mbp; *n* = 6 genes, 0.09 gene/Mbp), the most gene-rich chromosome in the human genome (Venter et al., 2001) (Supplementary Table S7). The highest number of eSNPs (23, including seven independent signals) was mapped for the *ZSCAN9* gene that has a potential function in the X-chromosome inactivation process (Luijk et al., 2018) (Supplementary Table S6A).

**FIGURE 2:**
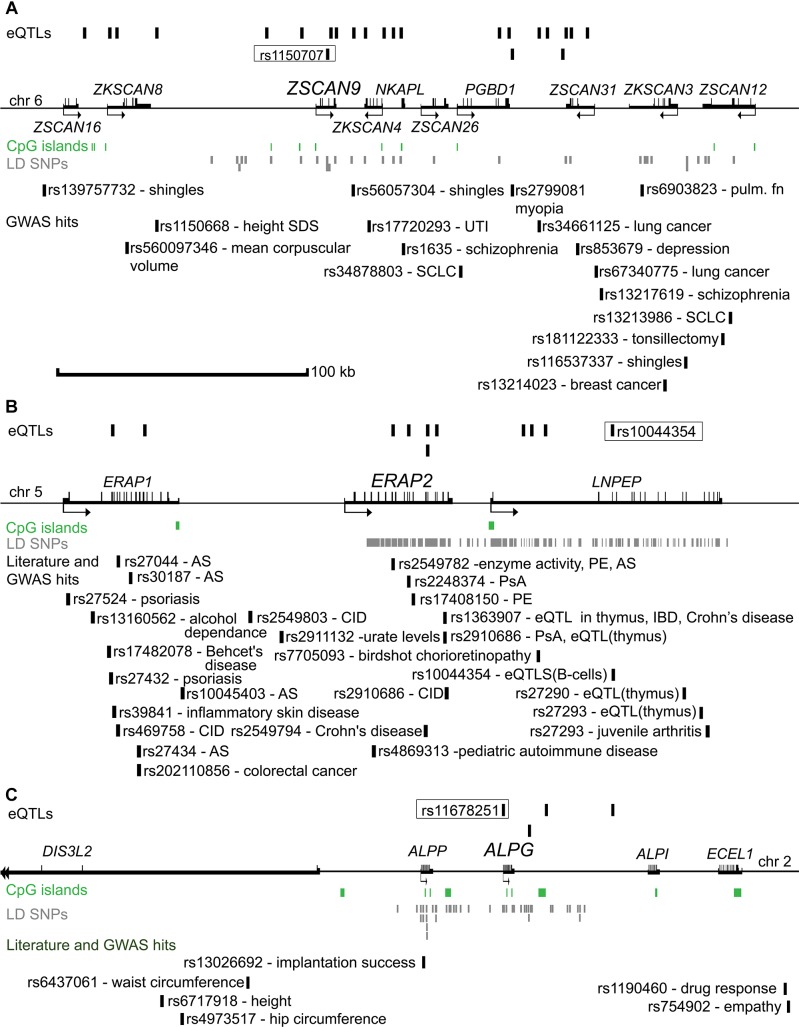
Genomic regions surrounding the eSNP-eGene pairs selected for validation **(A)** *ZSCAN9*, **(B)** *ERAP2*, and **(C)** *ALPG*. Distances between the genes are drawn in approximate scale and the eGene of the region is highlighted with an increased font. eSNPs identified in the discovery study are shown above the genes. The eSNP chosen for experimental validation is boxed and its proxy SNPs in LD (*r*^2^ > 0.8) are shown in gray below the genes. The landscape of reported genetic associations with common traits and diseases demonstrated at the bottom part of each subfigure was derived from the GWAS catalog and literature reports. The respective references are provided in Supplementary Table S9. *ALPG*, alkaline phosphatase, germ cell; *ALPI*, alkaline phosphatase, intestinal; *ALPP*, alkaline phosphatase, placental; AS, ankylosing spondylitis; chr, chromosome; CID, chronic inflammatory disease; *DIS3L2*, DIS3 like 3′-5′ exoribonuclease 2; *ECEL1*, endothelin converting enzyme like 1; eQTL, expression quantitative trait loci; *ERAP1*, Endoplasmic Reticulum Aminopeptidase 1; *ERAP2*, endoplasmic reticulum aminopeptidase 2; GWAS, genome-wide association study; IBD, inflammatory bowel disease; kb, kilobasepairs; LD, linkage disequilibrium; *LNPEP*, leucyl and cystinyl aminopeptidase; *NKAPL*, NFKB activating protein like; PE, preeclampsia; *PGBD1*, piggyBac transposable element derived 1; PsA, psoriatic arthritis; pulm. fn, pulmonary function; SCLC, Squamous cell lung carcinoma; SDS, standard deviation score; UTI, urinary tract infection; *ZKSCAN4*, zinc finger with KRAB and SCAN domains 4; *ZKSCAN8*, zinc finger with KRAB and SCAN domains 8; *ZSCAN9*, zinc finger and SCAN domain containing 9; *SCAN12*, zinc finger and SCAN domain containing 12; *ZSCAN16*, zinc finger and SCAN domain containing 16; *ZSCAN26*, zinc finger and SCAN domain containing 26; *ZSCAN31*, zinc finger and SCAN domain containing 31.

The associated eSNPs modulated the expression level of 63 eGenes expressed moderate to high in the human placenta (Supplementary Tables S6A,B and Figure 1C). The most significant placental eQTLs (FDR < 10^-5^) with a positive minor allele effect were modulating the expression of genes encoding the ribosomal protein RPL9 [fold change, fc(het) = 1.64, fc(min hom) = 2.32], the transcription factor ZSCAN9 [fc(het) = 3.62, fc(min hom) = 4.54] and the aminopeptidase ERAP2 [fc(het) = 3.22, fc(min hom) = 4.65] (Table 3). In the literature, genetic variants around *ERAP2* have been associated with autoimmunity related disorders, whereas the *ZSCAN9* gene is surrounded by the risk variants for neuropsychiatric diseases and cancer (Figure 2A,B).

**Table 3:** Top 10 protein coding eGenes from the discovery placental *cis*-eQTL analysis.

Lead eQTL chr:position	Alleles MAF	eGene (*n*, eQTLs)	*P*-value *P* (FDR)^a^	Read count^b^ (AA)	Read count (Aa)	Read count (aa)	fc^c^ (Aa)	fc (aa)	*R*^2^ for eQTL and gene expression^d^	eQTL in GTEx^e^	mRNA/protein^f^	Peng et al.
rs2249563 4:39453758	G > A 21%	*RPL9* (3)	1.09 × 10^-11^ 9.68 × 10^-7^	239 (180,317)	392 (304,454)	555 (singleton)	1.64	2.32	0.768	All tissues	All/all	Y
rs1150707 6:28229827^g^	C > T 30%	*ZSCAN9*^g^ (23)	1.17 × 10^-11^ 9.68 × 10^-7^	131 (92,230)	475 (267,686)	596 (493,644)	3.62	4.54	0.767	Skin	All/mixed	N
rs10044354 5:96984791^g^	C > T 41%	*ERAP2*^g^ (11)	8.63 × 10^-11^ 5.69 × 10^-6^	470 (339,1182)	1512 (813,2354)	2189 (1252,2602)	3.22	4.65	0.737	All tissues	Mixed/mixed	Y
rs10743750 12:31087400	C > T 28%	*DDX11* (2)	3.49 × 10^-10^ 1.92 × 10^-5^	503 (326,700)	355 (299,502)	182 (145,252)	0.71	0.36	0.713	All tissues	All/mixed	Y
rs7738394 6:150685788	T > C 30%	*PLEKHG1* (1)	4.34 × 10^-9^ 1.43 × 10^-4^	2079 (1602,2393)	1617 (1039,1784)	825 (755,895)	0.78	0.40	0.665	testis, esophagus	Enhanced (testis)/all	Y
rs7252798 19:21734730	C > T 46%	*ZNF100* (1)	4.57 × 10^-9^ 1.44 × 10^-4^	187 (119,280)	310 (201,480)	427 (304,588)	1.66	2.28	0.664	Most tissues	Mixed/NA	Y
rs567637 16:84500760	G > A 48%	*TLDC1* (5)	1.36 × 10^-8^ 4.07 × 10^-4^	1241 (1085,1837)	1886 (1157,2722)	2465 (1870,2956)	1.52	1.99	0.640	esophagus	Mixed/mixed	N
rs10865489 2:88147373	T > G 31%	*THNSL2* (2)	1.66 × 10^-8^ 4.57 × 10^-4^	577 (351,724)	319 (217,622)	171 (141,191)	0.55	0.30	0.636	All tissues	Enhanced (parathyroid)/mixed	Y
rs2612520 4:42645785	G > T 38%	*ATP8A1* (1)	2.86 × 10^-8^ 7.55 × 10^-4^	60 (25,263)	281 (42,816)	479 (280,821)	4.64	7.92	0.623	10 tissues adipose, breast etc.	Enhanced (parathyroid)/mixed	Y
rs11678251 2:232406577^g^	G > A 11%	*ALPG*^g^ (4)	7.62 × 10^-8^ 1.52 × 10^-3^	372 (33,834)	1336 (579,1708)	NA	3.60	NA	0.600	colon, lung	Enhanced (fallopian tube)/placenta	Y


*eQTL association testing was implemented in Matrix eQTL (Shabalin, 2012) using linear regression adjusted by the pregnancy outcome (normal term or PE, GD, SGA, LGA pregnancy), labor activity and newborn sex. ^*a*^False discovery rate was calculated according to Benjamini and Hochberg method. ^*b*^Normalized read count, median (min, max). ^*c*^Fold change from median normalized read count of major homozygotes. ^*d*^Fraction of gene expression variation explained by the eQTL genotypes (range 0–1). ^*e*^GTEx reports eQTL data for 48 tissues, excluding placenta (https://gtexportal.org). ^*f*^The Human Protein Atlas (https://www.proteinatlas.org/). ^g^Selected for validation. *ALPG*, alkaline phosphatase, germ cell; *ATP8A1*, ATPase phospholipid transporting 8A1; chr, chromosome; *DDX11*, DEAD/H-box helicase 11; *DNAJC15*, DnaJ heat shock protein family (Hsp40) member C15; *ERAP2*, endoplasmic reticulum aminopeptidase 2; fc, fold change; MAF, minor allele frequency; NA, not available; *PLEKHG1*, pleckstrin homology and RhoGEF domain containing G1; ptg, parathyroid gland; *RPL9*, ribosomal protein L9; *THNSL2*, threonine synthase like 2; *TLDC1*, TBC/LysM-associated domain containing 1; *ZNF100*, zinc finger protein 100; *ZSCAN9*, zinc finger and SCAN domain containing 9.*

The largest eSNP effect was detected for the phospholipid transporter *ATP8A1* transcript levels [FDR = 7.44 × 10^-4^; fc(het) = 4.64, fc(min hom) = 7.92]. Overall, the number of eQTLs with a positive effect on gene expression was twice as many compared to those with a negative effect. Top eQTLs exhibiting negative minor allele effect were associated with the expression of *DDX11, PLEKHG1*, and *THNSL2* implicated in fetal development, signaling and cellular proliferation, respectively (Table 3). Nearly 80% of the identified eGenes (*n* = 50) overlapped with the gene list reported by Peng et al. (Figure 1D). Despite the differences in the study design, ten of the 63 identified placental eGenes in our dataset fall within 1% and 45 genes within 25% of the loci with the lowest *P*-value in the previous independent dataset (Peng et al., 2017; Supplementary Tables S6B,C). As an added value we identified 13 novel placental eGenes (10 protein coding, 3 pseudogenes), including nine associations that were supported by the GTEx data in other tissues (Table 4). Amongst these, the identified eQTLs for the genes *ZSCAN9* and *TLDC1* were ranked within the top 10 gene expression associated variants in our analysis (Table 3). The expression level of *RBPJ, TCIM, TPRN*, and *THUMPD2* was associated with potential placenta specific eQTLs that have not been detected in the GTEx project.

**Table 4:** Characteristics of 13 novel placental eGenes, identified in the current study.

Lead *cis*-eSNP Chr: Position	Alleles MAF	eGene (number of *cis*-eSNPs)	*P*-value*P* (FDR)^a^	Read count^b^			Fold change^c^		*R*^2^ for eQTL and gene expression^d^	Biological function	mRNA/protein^e^	GTEx^f^
	
				Maj hom	Het	Min hom	Het	Min hom				
rs1150707 chr6:28229827	T < C 0.30	*ZSCAN9* (23)	1.17 × 10^-11^ 9.68 × 10^-7^	131	475	596	3.62	4.54	0.77	Transcription factor^g^	All/mixed	Y
rs1132812 chr16:30186830	A < G 0.45	*SMG1P5* (2)	2.93 × 10^-9^ 1.02 × 10^-4^	145	440	610	3.03	4.21	0.67	Pseudogene	NA/NA	Y
rs567637 chr16:84500760	A < G 0.48	*TLDC1* (5)	1.36 × 10^-8^ 4.07 × 10^-4^	1242	1886	2465	1.52	1.99	0.64	Cell proliferation (Nguyen et al., 2018)	Mixed/all	Y
rs9320475 chr6:113922342	T < C 0.21	*FO393415.1* (1)	5.34 × 10^-8^ 1.26 × 10^-3^	91	241	255	2.65	2.80	0.61	Pseudogene	NA/NA	Y
rs3810756 chrX:119417694	T < C 0.05	*SLC25A43* (2)	3.67 × 10^-7^ 4.48 × 10^-3^	192	131	123	0.68	0.64	0.56	Mitochondrial transporter (Gabrielson et al., 2016)	Mixed/enhanced (liver)	Y
rs10767971 chr11:32874118	T < C 0.35	*PRRG4* (4)	5.64 × 10^-7^ 6.45 × 10^-3^	1023	1400	1923	1.37	1.89	0.55	Neuronal regulation (Justice et al., 2017)	Mixed/mixed	Y
rs1053846 chr1:39757468	G < T 0.21	*PPIE* (3)	1.29 × 10^-6^ 1.24 × 10^-2^	199	306	394	1.53	1.98	0.52	Splicing (Chanarat and Sträßer, 2013)	All/all	Y
rs2871198 chr4:26290933	C < T 0.33	*RBPJ* (1)	1.91 × 10^-6^ 1.68 × 10^-2^	2663	3105	3703	1.17	1.39	0.51	Transcriptional regulation (Han et al., 2002)	All/NA	N
rs7046565 chr9:109539940	C < T 0.43	*YBX1P6* (1)	6.26 × 10^-6^ 3.22 × 10^-2^	32	156	164	4.87	5.15	0.48	Pseudogene	NA/NA	Y
rs10869496 chr9:75085168	A < C 0.36	*NMRK1* (1)	7.20 × 10^-6^ 3.52 × 10^-2^	433	353	261	0.82	0.60	0.47	NAD metabolism (Bieganowski and Brenner, 2004)	All/mixed	Y
rs4370521 chr8:40111873	G < A 0.33	*TCIM* (1)	7.63 × 10^-6^ 3.62 × 10^-2^	300	473	631	1.58	2.10	0.47	Cell cycle regulator (Jung et al., 2006)	Mixed/mixed	N
rs7850758 chr9:137293773	G < A 0.06	*TPRN* (1)	1.29 × 10^-5^ 4.69 × 10^-2^	218	304	NA	1.40	NA	0.45	Hearing (Li et al., 2010)	All/all	N
rs11692913 chr2:39853203	A < G 0.35	*THUMPD2* (1)	1.44 × 10^-5^ 4.77 × 10^-2^	217	230	281	1.06	1.29	0.45	tRNA metabolism^g^	Mixed/mixed	N


*eQTLs were tested using linear regression adjusted by the pregnancy outcome (normal term or PE, GD, SGA, LGA pregnancy), labor activity and newborn sex. ^*a*^False discovery rate was calculated according to Benjamini and Hochberg method. ^*b*^Normalized read count, median (min, max). ^*c*^Fold change compared to median normalized read count of major homozygotes. ^*d*^Fraction of gene expression variation explained by the *cis*-eSNP genotypes (scale 0–1). ^*e*^Data from the Human Protein Atlas (https://www.proteinatlas.org/). ^*f*^eQTL identified in GTEx for at least one tissue. ^*g*^Predicted annotation from UniprotKB. Maj hom, major homozygote; Het, heterozygote; Min hom, Minor homozygote; *NMRK1*, nicotinamide riboside kinase 1; *PPIE*, peptidylprolyl isomerase E; *PRRG4*, proline rich and Gla domain 4; *RBPJ*, Recombination signal binding protein for immunoglobulin kappa J region; *SLC25A43*, solute carrier family 25 member 43; *SMG1P5*, SMG1 pseudogene 5; *TCIM*, transcriptional and immune response regulator; *THUMPD2*, THUMP domain containing 2; *TLDC1*, TBC/LysM-associated domain containing 1; *TPRN*, taperin; *YBX1P6*, Y-box binding protein 1 pseudogene 6; *ZSCAN9*, Zinc finger and SCAN domain containing 9.*

### Placental eQTLs and Risk to Term Pregnancy Complications

In the discovery sample, none of the placental eQTLs were significantly associated with adverse pregnancy outcomes at term after applying multiple testing correction (Supplementary Table S6D). Among nominal suggestive associations a substantial effect of *ZSCAN9* eSNP (rs1150707) was detected to the risk of GD (logistic regression; OR = 21, *P* = 0.02) or the birth of a SGA newborn (OR = 15.4, *P* = 0.04). This was not confirmed in the analysis of the full REPROMETA sample set (GD: OR = 1.53, *P* = 0.14; SGA: OR = 1.18, *P* = 0.45).

Additional suggestive association with GD was detected for the eSNP of *PLEKHG1* (rs7738394 OR = 0.05, *P* = 0.02), previously linked to the risk of preeclampsia (Gray et al., 2018). An eSNP modulating the expression of *DNAJC15* was nominally associated with LGA (rs17553284; OR = 0.05, *P* = 0.02). Interestingly, this variant has been reported as a top signal driving differentiated allele-specific expression between African and European populations (Tian et al., 2018).

### Placental eGenes Exhibit Broad Functional Portfolio

According to the Human Protein Atlas, the identified 63 eGenes exhibited mostly mixed or ubiquitous gene and protein

expression (Figure 1E). Only few genes are specifically expressed in the placenta, e.g., placental alkaline phosphatase *ALPG* and *PSG7* (Supplementary Tables S6A,B). Based on the available literature evidence, the placental eGenes represent a broad portfolio of functional categories (Table 5). Almost 1/3 of eGenes were associated with general cellular functions [structure (*n* = 10, e.g., *DCTN5, TTLL4*) and transport (*n* = 10, e.g., *AQP11, SLC27A6*)]. About 10% of genes are implicated in the immune function and immune-defense mechanisms that are critical in maintaining healthy pregnancy (*n* = 6, e.g., *ERAP2, TRIM5*). A functional enrichment analysis of eGenes highlighted 35 Gene Ontology (GO) categories (Supplementary Table S6E). Interestingly, the most significantly enriched pathways were “ruffle membrane” (*IFIT5, ADAM17, EPB41L5*, FDR = 1.81 × 10^-2^) contributing to the formation of motile cell surface and “ATPase activity, coupled” (*ATP1A4, DDX11, RFC3, ATP8A1, PEX6*; FDR = 2.88 × 10^-2^), critical for the active transport of molecules across cell membrane. Among the significantly (FDR < 0.05) enriched GO categories there were also several pathways implicated in development, signaling and immune function.

**Table 5:** Identified eGenes classified by their main functional category.

Function	*n*	Genes
Cellular transport	10	*AQP11, ATP1A4, ATP8A1, CNIH4, HEATR5A, SLC25A43, SLC27A6, SLC44A1, SNX25, TMC4, DNAJC15*
Cell structure	10	*CEP128, CEP72, DCTN5, EPB41L5, FAM118A, HEATR4, LYPD5, NEO1, PEX6, TPRN, TSGA10, TTLL4*
Transcription	7	*TCIM, TRIM66, RBPJ, ZNF100, ZNF266, ZNF749, ZSCAN9*
Immunity/defense	6	*CBLB, ERAP2, IFIT5, IL36RN, PSG7, TRIM5*
Enzymatic activity	5	*ALPG, ATG10, IP6K3, NMRK1, THNSL2*
DNA replication/repair	4	*DDX11, RAD52, RFC3, CYREN*
Translational regulation and protein modification	4	*SPSB2, RPL9, PPIE, THUMPD2*
Signaling	4	*ADAM17, GLS, PLEKHG1, PRRG4*
Cellular proliferation and differentiation	2	*TLDC1, SLFN5*
Other	4	*ABHD11, PSMD5, THNSL2, WDR91*
Unclassified	1	*HEATR5A*
Pseudogene	5	*FO393415, GUCY1B2, YBX1P6, SMG1P5, HTR7P1*


*Genes were grouped according to data retrieved from UniProtKB/Swiss-Prot database, Entrez gene database and/or literature.*

To obtain more potential functional insight, the identified eSNPs and eGenes were investigated for the overlap with association signals from published GWAS. Six placental eSNPs have been directly associated with either blood metabolite levels [eSNP-eGenes pairs: rs2576452-*TMC4*, rs2041073-*HEATR4* (Shin et al., 2014), rs6743376-*IL36RN* (Matteini et al., 2014)], risk to neurodegenerative diseases [rs10767971-*PRRG4* (Latourelle et al., 2009), rs1129187-*PEX6* (Jun et al., 2016)] or cancer [rs12309274-*RAD52* (Zhang et al., 2014)] (Supplementary Table S6F). These eSNP-eGene pairs have also been reported in GTEx with an effect in at least one other tissue. However, there was no statistically significant enrichment of GWAS hits among the identified eSNPs (6 of 199) compared to the overall proportion of GWAS SNPs (11,475 of 353,599) among the tested variants (χ^2^ test: OR = 0.93 [0.41 – 2.09], *P* = 0.85). In total 58 of 63 of the eGenes were located within 100 kb of various reported GWAS loci and 45 of them were assigned as the closest gene (Supplementary Table S6G). However, due to the abundance of the GWAS associated genes (GWAS Catalog: 7433 of 11,733 genes tested in this study), this does not represent a significant enrichment (OR = 1.44 [0.84 – 2.50], *P* = 0.19).

### Validation of the eSNPs of *ALPG, ERAP2*, and *ZSCAN9* Using Taqman RT-qPCR

**FIGURE 3:**
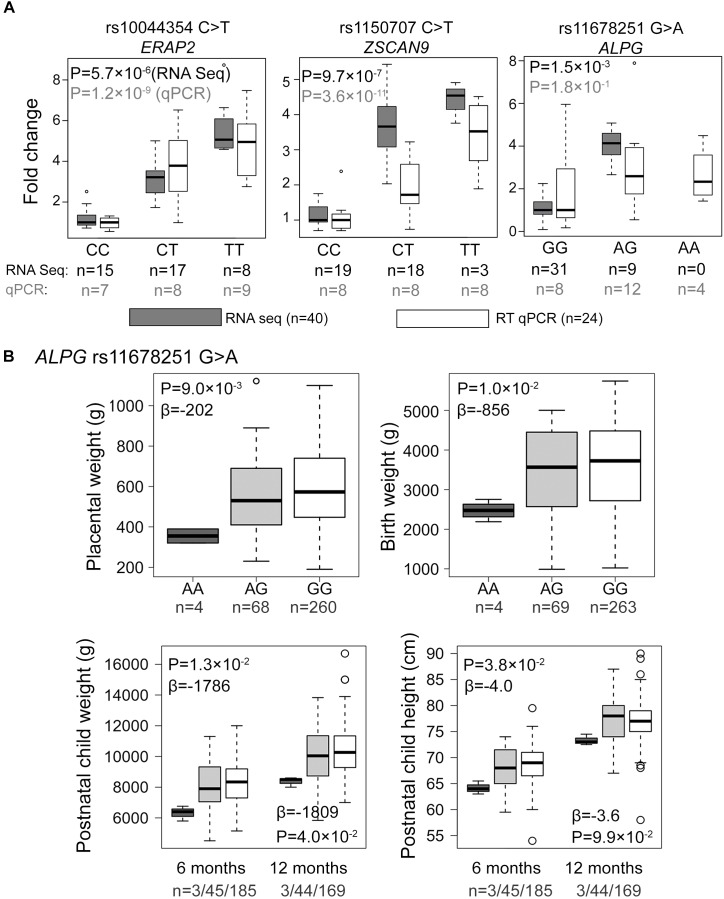
Experimental validation and genetic association testing with newborn parameters. **(A)** Comparative effect sizes from the RNA-Seq discovery and Taqman RT qPCR experiments for the eSNP-eGene associations selected for the experimental validation. For the estimation of fold changes, median expression level in the placentas with the major homozygote genotype was considered as the reference. Statistical analysis for association testing was performed under linear regression using additive model adjusted by the newborn sex, pregnancy complication group and labor activity. As the validation dataset was not matched for gestational age at delivery, this parameter was additionally incorporated as a covariate in the validation step. The shown *P*-values have been corrected multiple testing using FDR method. *n*, number of samples. **(B)** Effect of the *ALPG* c.-318 G > A (rs11678251) eSNP on the offspring growth parameters at birth and during infancy in the REPROMETA dataset. The data was available for 336 newborns at delivery, and their follow-up data at the of 6 (*n* = 233) and 12 (*n* = 216) months of age. Genetic association testing was performed using linear regression under recessive model adjusted by fetal sex. In testing the newborn parameters, gestational age at delivery was used as an additional covariate. The obtained nominal *P*-values < 0.05 were considered as supportive for the trend of a tested association. Beta values reflect the estimated effect of the AA-homozygosity on the tested parameter.

From amongst the most significant eQTL signals, three (*ZSCAN9* c.568+1990 C > T, *ERAP2* g.96984791 C > T, *ALPG* c.-318 G > A) were selected for validation experiments in an extended set of genotyped REPROMETA placental samples (*n* = 24/gene; see section “Materials and Methods”; Supplementary Table S2). In order to clarify potential eSNP effects on the neighboring genes, the analysis included also the *ALPP* gene that represents a duplicate locus to *ALPG*, and the *ERAP1* and *LNPEP* genes flanking *ERAP2* (Figure 2). The eQTL effect on expression levels of *ZSCAN9* (linear regression, additive model FDR = 3.6 × 10^-11^) and *ERAP2* (FDR = 1.2 × 10^-9^) were robustly validated (Figure 3A and Supplementary Table S8). The expression of *ERAP1* and *LNPEP* was not modulated by *ERAP2* g.96984791 C > T. Although in the validation dataset the effect of *ALPG* c.-318 G > A on *ALPG* expression level did not reach statistical significance (FDR = 0.18), the placentas carrying the minor A-allele exhibited a trend for increased *ALPG* and decreased *ALPP* expression (*ALPP/ALPG* ratio FDR = 1.2 × 10^-2^; Supplementary Table S8). This observation requires further confirmation as there is a substantial difference between the expression levels of *ALPP* and *ALPG* that may have led to a statistical artifact.

### Suggestive Association of the *ALPG* -318 G > A Variant With Newborn Growth Parameters in the REPROMETA Study

Association testing targeting the highlighted eSNPs near *ZSCAN9, ERAP2*, and *ALPG* was carried out with newborn parameters in the full REPROMETA sample set (*n* = 336) applying linear regression (Table 2). None of the tested associations resulted in a statistically significant outcome after correction for multiple testing. However, the *ALPG* c.-318 G > A exhibited a suggestive association under recessive model with the placental weight (AA-genotype effect -202 g, nominal *P* = 9.3 × 10^-3^), as well as newborns’ chest circumference (-2.1 cm, *P* = 3.2 × 10^-3^) and weight (-856 g, *P* = 1.0 × 10^-2^) (Figure 3B and Table 6). Supportingly, previous studies have associated SNPs near *ALPG* with other anthropometric traits (height, waist and hip circumference) and implantation success (Figure 2C). Also, *ZSCAN9* c.568+1990 C > T showed a nominal association with placental weight (TT-homozygotes: -54 g, *P* = 4.4 × 10^-2^).

**Table 6:** Association testing of validated eQTL with newborn traits in the REPROMETA study (*n* = 335).

Tested parameter and genetic model	*ALPG*		*ERAP2*		*ZSCAN9*	
			
	G > A rs11678251		C > T rs10044354		C > T rs1150707	
			
	Effect (SE)	*P*-value	Effect (SE)	*P*-value	Effect (SE)	*P*-value
**Placental weight (g)**						
Additive	-48 (19)	**1.0 × 10**^-^**^2^**	6 (12)	6.1 × 10^-1^	-12 (13)	3.4 × 10^-1^
Recessive	-202 (77)	**9.0 × 10**^-^**^3^**	-3 (21)	8.9 × 10^-1^	-54 (27)	**4.4 × 10**^-^**^2^**
**Birth weight (g)**						
Additive	-161 (81)	**4.7 × 10**^-^**^2^**	42 (50)	4.1 × 10^-1^	-59 (55)	2.8 × 10^-1^
Recessive	-856 (332)	**1.0 × 10**^-^**^2^**	64 (92)	4.9 × 10^-1^	-145 (116)	2.1 × 10^-1^
**Birth length (cm)**						
Additive	-0.4 (0.3)	1.4 × 10^-1^	0.2 (0.2)	3.0 × 10^-1^	-0.2 (0.2)	4.3 × 10^-1^
Recessive	-1.9 (1.2)	1.2 × 10^-1^	0.4 (0.3)	2.7 × 10^-1^	-0.4 (0.4)	4.1 × 10^-1^
**Head circumference at birth (cm)**						
Additive	-0.3 (0.2)	2.0 × 10^-1^	0.1 (0.1)	3.4 × 10^-1^	-0.3 (0.1)	7.2 × 10^-2^
Recessive	-1.1 (0.9)	2.0 × 10^-1^	0.2 (0.2)	3.4 × 10^-1^	-0.5 (0.3)	1.2 × 10^-1^
**Chest circumference at birth (cm)**						
Additive	-0.3 (0.2)	1.6 × 10^-1^	0.3 (0.2)	1.0 × 10^-1^	-0.1 (0.2)	5.1 × 10^-1^
Recessive	-2.1 (0.7)	**3.2 × 10**^-^**^3^**	0.3 (0.4)	3.7 × 10^-1^	-0.4 (0.4)	3.2 × 10^-1^
**At 6 months of age (only recessive model)^a^**						
Weight	-1786 (711)	**1.3 × 10**^-^**^2^**	120 (206)	5.6 × 10^-1^	169 (263)	5.2 × 10^-1^
Height	-4.0 (1.9)	**3.8 × 10**^-^**^2^**	0.4 (0.6)	5.0 × 10^-1^	0.2 (0.7)	8.4 × 10^-1^
**At 12 months of age (only recessive model)^a^**						
Weight	-1809 (876)	**4.0 × 10**^-^**^2^**	-85 (262)	7.5 × 10^-1^	45.2 (330.3)	8.9 × 10^-1^
Height	-3.6 (2.2)	9.9 × 10^-2^	0.5 (0.7)	4.2 × 10^-1^	0.27 (0.82)	7.4 × 10^-1^


*Association testing with birth parameters was implemented using linear regression analysis under additive and recessive model, adjusted by newborn sex and gestational as covariates. The obtained nominal *P*-values < 0.05 (in bold) were considered as supportive for the trend of a tested association. The Bonferroni corrected statistical significance level was estimated *P* < 1.2 × 10^-*3*^. ^*a*^Postnatal growth data was available for 231 children (121 girls and 110 boys). Based on the outcome of the primary analysis with birth parameters, only recessive model (sex-adjusted) was applied for association testing with postnatal growth. *ALPG*, alkaline phosphatase, germ cell; *ERAP2*, endoplasmic reticulum aminopeptidase 2; *ZSCAN9*, zinc finger and SCAN domain containing 9.*

The eSNPs were further tested for the association with the growth of the REPROMETA children during their first year of life. Based on the outcome of the association testing with birth parameters, only the effect of homozygosity (recessive model) was applied. Six and twelve months old infants who were homozygous for the *ALPG* c.-318 A-allele had maintained lower weight compared to alternative genotype carriers (effect at 6 m: -1786 g, nominal *P* = 1.3 × 10^-2^; 12 m: 1809 g, *P* = 4.0 × 10^-2^; Figure 3B). Also, their height remained lower during 6 postnatal months (effect -4.0 cm, *P* = 3.8 × 10^-2^), but it was caught up by the 1st birthday (*P* > 0.05). eSNPs of *ERAP2* and *ZSCAN9* showed no association with infant growth parameters.

### Meta-Analysis of *ALPG* -318 G > A in the HAPPY PREGNANCY and ALSPAC Cohort Samples

Association testing of the *ALPG* c.-318 G > A variant with the newborn anthropometric parameters was extended to two population-based cohorts HAPPY PREGNANCY (Estonia, *n* = 408) and ALSPAC (United Kingdom, *n* = 7669). The analysis in the individual cohorts and the meta-analysis combining the results from three studies did not replicate the initial associations with newborns’ and placental weight detected in the REPROMETA study (Table 7). However, only in the meta-analysis the association of *ALPG* c.-318 G > A with the newborn’s smaller head circumference reached nominal statistical significance (*P* = 0.042; effect -0.4 cm). The effect of the AA-genotype across the studies varied from -0.3 to -1.1 cm.

**Table 7:** Meta-analysis of the genetic association testing of the *ALPG* c.-318 G > A (rs11678251) with newborn traits in the REPROMETA, HAPPY PREGNANCY, and ALSPAC studies.

Newborn parameter	REPROMETA			HAPPY PREGNANCY			ALSPAC			Meta-analysis		
				
	Effect (SE)	*P*-value	*n*	Effect (SE)	*P*-value	*n*	Effect (SE)	*P*-value	*n*	Effect (SE)	*P*-value	*n*
*All newborns*												
Birth weight (g)	-856 (332)	**1.0 × 10**^-^**^2^**	335	-199 (164)	2.3 × 10^-1^	408	-61 (57)	2.9 × 10^-1^	7669	-96 (53)	7.2 × 10^-2^	8412
Placental weight (g)	-202 (77)	**9.0 × 10**^-^**^3^**	331	-19 (46)	6.8 × 10^-1^	406	29 (27)	2.9 × 10^-1^	3123	-2 (22)	9.2 × 10^-1^	3860
Birth length (cm)	-1.9 (1.2)	1.2 × 10^-1^	330	-0.7 (0.7)	3.3 × 10^-1^	390	-0.3 (0.3)	3.6 × 10^-1^	6077	-0.4 (0.3)	1.2 × 10^-1^	6797
Head circumference (cm)	-1.1 (0.9)	2.0 × 10^-1^	328	-0.8 (0.5)	1.1 × 10^-1^	343	-0.3 (0.2)	1.8 × 10^-1^	6159	-0.4 (0.2)	**4.2 × 10**^-^**^2^**	6830
Chest circumference (cm)	-3.53 (1.25)	**5.1 × 10**^-^**^3^**	325	-0.86 (0.7)	2.2 × 10^-1^	337	NA	NA	NA	-1.49 (0.61)	**1.5 × 10**^-^**^2^**	662


*Linear regression analysis under recessive model adjusted by covariates newborn sex and gestational age (in days for REPROMETA and HAPPY PREGNANCY, in weeks for ALSPAC) was applied. The obtained nominal *P*-values < 0.05 (in bold) were considered as a suggestive association. Bonferroni significance level corrected for the number of individual tests was calculated 0.05/14 = 3.6 × 10^-*3*^. *ALPG*, alkaline phosphatase, germ cell; NA, not available; SE, standard error.*

## Discussion

Genetic variants modulating human placental gene expression have been understudied and so far only one genome-wide analysis of placental eQTLs has been published with no external independent validation (Peng et al., 2017). The current report represents the second study addressing specifically the landscape of placental *cis*-eQTLs, including experimental validation of selected top eSNP-eGene pairs and further exploration of their potential effect on human traits. The study identified 88 independent eSNP signals modulating the expression of 63 placental genes (termed as eGenes). Importantly, 50 loci overlapped with the placental eGene list from Peng et al. (2017) and thus, can be claimed as robust placental eQTL signals to be explored further for their effect on placental function, fetal development, neonatal outcomes and postnatal disease risks (Supplementary Table S6B). Unfortunately, the direction of these eSNP effects on the expression of the eGenes could not be compared among the studies as the reference allele was not equivocally stated in Peng et al. (2017).

The current study identified additional 13 novel placental eGenes (Table 4). Among these, *ZSCAN9* represented an interesting novel locus modulated by several linked genetic variants and their highly significant eQTL effect was confirmed in experimental validation (Figure 3A, Table 3, and Supplementary Table S6A). A recent study reported that *ZSCAN9 cis*-eQTLs exhibit female specific effects on human X-chromosomal methylation (Luijk et al., 2018). The ancestral A-allele of the variant rs1736891 was associated *in cis* with hypomethylated CpGs and high *ZSCAN9* expression, and *in trans* with hypomethylation of the CpG islands near X-chromosomal genes variably escaping X-chromosomal inactivation. Also, in the current study, rs1736891 was detected among the top placental eQTLs associated with the *ZSCAN9* transcript levels (Supplementary Table S6A). The placenta is well known for its unusual hypomethylated epigenome and thus, the *ZSCAN9* eSNPs may further contribute to the organ-specific enhanced expression of X-chromosomal genes that escape inactivation. As an additional interesting observation from the current study, worth to be followed-up in a larger cohort, was a potential effect of *ZSCAN9* eQTLs to the risk of GD. Other interesting placental eSNP-eGene pairs are rs2871198-*RBPJ* and rs4370521-*TCIM* that have not been reported in GTEx. TCIM is a positive regulator of the Wnt/beta-catenin pathway (Jung et al., 2006) and RBPJ regulates the transcription of the Notch signaling pathway genes via recruitment of chromatin remodeling complexes to its targets (Han et al., 2002). Both pathways are critical in early human development.

When comparing the current placental eSNP analysis outcome with the previous study (Peng et al., 2017), the number of identified eGenes appears not as extensive (*n* = 63 vs. 3218; Supplementary Table S6C). However, in order to minimize false-positive predictions and to avoid spurious associations that are common in eQTL analysis (Huang et al., 2018), several stringent criteria for the eQTL detection were applied. One core difference between the two studies was in defining *cis*-eQTLs (±100 kbp vs. ±500 kbp from gene), resulting in a five-fold smaller proportion of the genome targeted to the *cis*-eQTL testing compared to the previous study (Peng et al., 2017). Another critical aspect was in applying a more conservative statistical significance threshold for claiming eQTL effect (5% vs. 10% FDR) that further reduced the number of robust claims. Thirdly, while this study relied only on directly genotyped genetic variants (*n* = 661,354), the previous placental eQTL analysis included also imputed SNPs (*n* = 5,748,854) that possibly increased the number of identified associations. Taken together, the careful study design facilitated the detection of high-confidence placental eSNPs, the majority of those were confirmed either by the independent dataset (Peng et al., 2017) or by the experimental approach. However, it has to be acknowledged that as the number of analyzed samples was modest [*n* = 40 vs. 199 (Peng et al., 2017)], this possibly affected the study power.

A critical role of placental function in fetal programming and its further impact on the health across the life span has been proposed already several years ago (Bonnin et al., 2011; Longtine and Nelson, 2011; Kwon and Kim, 2017). As an example, the two haplotype variants of the placenta-specific *GH2* gene encoding placental growth hormone have been associated with the programming of adult height (Timasheva et al., 2013). In the current study, six eSNPs represented directly GWAS hits (6.8% of independent eQTL signals) and 45/63 eGenes were assigned as the closest gene to various GWAS SNPs for biomedical traits or diseases (Supplementary Table S6G and Figure 2). However, we were not able to confirm a significant enrichment of GWAS top hits among the detected placental eSNP and eGenes that was reported by the previous placental eQTL study (Peng et al., 2017).

Altered placental gene expression and the malfunctioning placenta are well-acknowledged risk factors for pregnancy complications and impaired fetal development (Sõber et al., 2015, 2016; Ashar-Patel et al., 2017; Sundrani et al., 2017; Sifakis et al., 2018). Our pilot analysis in the REPROMETA study detected a suggestive association of the AA-genotype of the eSNP *ALPG* c.-318 (rs11678251) with the reduced weight of the placenta, newborn, and infant until 1 year of age (Figure 3B and Table 6). *ALPG* represents one of the core trophectoderm (TE)-specific genes that drives the first cellular differentiation to inner cell mass and TE in early human development (Bai et al., 2012). However, the identified associations were not replicated in two independent and larger pregnancy cohorts of the HAPPY PREGNANCY and ALSPAC studies (Table 7). As the number of common genetic (and non-genetic) factors affecting anthropometric parameters is considered high and the effect sizes of individual variants are expected to be small (Wood et al., 2014; Tachmazidou et al., 2017), even a larger dataset than utilized in the current meta-analysis (*n* = 8412) would be required to confirm or reject the preliminary findings.

In summary, the study robustly demonstrated the role of genetic variation in driving the transcriptome profile of the human placenta and emphasized the importance to explore further the link between placental eQTLs, prenatal developmental programming and susceptibility to complex diseases.

## Data Availability

The raw data supporting the conclusions of this manuscript will not be made publicly available to protect participant confidentiality. Clinical RNA-seq data will be made available by the authors upon request to any qualified researcher, pending ethical approval.

## Ethics Statement

The REPROgrammed fetal and/or maternal METAbolism (REPROMETA) and HAPPY PREGNANCY (full study name “Development of novel non-invasive biomarkers for fertility and healthy pregnancy”) studies were approved by the Ethics Review Committee of Human Research of the University of Tartu, Estonia (Permissions Nos. 146/18, 27.02.2006; 150/33, 18.06.2006; 158/80, 26.03.2007; 221/T-6, 17.12.2012; and 286/M-18, 15.10.2018). All study participants were recruited, and the study material was collected at the Women’s Clinic of Tartu University Hospital, Estonia in 2006–2011 (REPROMETA) and in 2013–2015 (HAPPY PREGNANCY). All participants in the REPROMETA and HAPPY PREGNANCY studies were of white European ancestry and living in Estonia. A written informed consent to participate in the study was obtained from each individual prior to recruitment. Ethical approval for the ALSPAC was obtained from the ALSPAC Ethics and Law Committee and the Local Research Ethics Committees. The study recruited pregnant women in Bristol area, United Kingdom in 1991–1992 (http://www.alspac.bris.ac.uk). Consent for biological samples was collected in accordance with the Human Tissue Act (2004). Informed consent for the use of data collected via questionnaires and clinics was obtained from participants following the recommendations of the ALSPAC Ethics and Law Committee. All procedures and methods in the three studies have been carried out in compliance with the guidelines of the Declaration of Helsinki.

## Author Contributions

ML conceived the study. TK, KR, and ML designed the analyses and experiments. RF and ML provided the study materials. KR coordinated the clinical phenotyping and sampling in Estonia. TK conducted the experiments. TK and RB analyzed the data. TK, KR, RB, RF, and ML interpreted the data. TK and ML wrote the manuscript. All authors contributed to critical reading and commenting of the manuscript, and final approval of manuscript.

## Conflict of Interest Statement

The authors declare that the research was conducted in the absence of any commercial or financial relationships that could be construed as a potential conflict of interest.

## Footnotes

1 http://www.alspac.bris.ac.uk

2 http://www.ensembl.org/index.html

3 http://fuma.ctglab.nl/

4 https://www.ebi.ac.uk/gwas/docs/file-downloads, all associations v1.0_e91

5 http://pngu.mgh.harvard.edu/~purcell/plink/
