# Supplementary material for: The Effect of Genetic Variation on the Placental Transcriptome in Humans
Source: Front Genet. 2019 Jun 11;10:550. doi: 10.3389/fgene.2019.00550 (PMC6581026; doi:10.3389/fgene.2019.00550)
Supplement: Supplementary file 1 [file Data_Sheet_1.pdf]

List of Supplementary Materials

Supplementary Methods

Supplementary Table S1 Primer sequences for genotyping using Sequenom iPLEX Gold platform.

Supplementary Table S2 Clinical parameters of the REPROMETA cases in the placental *cis*-eQTL discovery and in the validation of the *cis*-eSNP/eGene pair.

Supplementary Table S3 Taqman Assay probes for gene expression quantification with RT-qPCR.

Supplementary Table S4 Allele and genotype frequencies of SNPs taken forward to the genetic association testing with newborn parameters.

Supplementary Table S5 Growth parameters of REPROMETA children at 6 and 12 months of age.

Supplementary Table S6 All placental eQTLs identified in the discovery analysis (separate .xlsx file)

Supplementary Table S7 Chromosomal localization of identified *cis*-eSNPs/eGene pairs.

Supplementary Table S8 Results of eQTL validation with Taqman RT-qPCR in placental samples from REPROMETA (n=24 per SNP).

Supplementary Table S9 Scientific literature data for the *ALPG*, *ALPP*, *ERAP2*, *ERAP1*, *LNPEP* and *ZSCAN9* genes.

Supplementary Figure S1 Workflow of the eQTL identification and validation.

Supplementary Figure S2 Chromosomal localization and gene expression level of eGenes.

Supplementary references

Supplementary Data 1. The results of eQTL testing for all analyzed SNPs (separate .zip file). Association between gene expression level (normalized read counts) and SNPs (±100kb from gene coordinates) was tested using linear regression adjusted by the pregnancy outcome, labor activity and newborn sex. The analysis was implemented in the Matrix eQTL platform (Shabalin, 2012).

Supplementary Methods

REPROMETA clinical subgroup definitions

The REPROMETA study represents family trios (mother, father, placenta) recruited before or shortly after delivery of a singleton newborn at the Women's Clinic of Tartu University Hospital, Estonia. The study was designed to include well-defined, clinically diagnosed diverse scenarios of pregnancy outcomes at term (gestational age 36-42 weeks) along with the control group. The full REPROMETA placental sample set analyzed in the current study (n=336) is comprised of five clinical subgroups: delivery of a small-for-gestational-age (SGA, birth weight <10th centile based on data from Estonian Medical Birth Registry (Sildver et al., 2015); n=65) or large-for-gestational-age newborn (LGA, >90th centile; n=83), cases of maternal gestational diabetes (GD; n=41) or severe late-onset preeclampsia (PE; n=43), normal term pregnancies (birth weight 10th-90th centile; n=104). SGA and LGA newborns were defined on the basis of Estonian Medical Birth Registry (Sildver et al., 2015). All PE cases represented the severe form of preeclamptic pregnancies and were defined as hypertensive (systolic blood pressure ≥160mmHg and/or diastolic blood pressure ≥110mmHg) and had proteinuria of ≥5g in 24 hours or neurological symptoms (Brown et al., 2018). GD was diagnosed when 75g oral glucose tolerance test (OGTT) performed at 24–28 weeks of gestation revealed either a fasting venous plasma glucose level of ≥5.1 mmol/l and/or at 1h and 2h later plasma glucose level of ≥10.0 mmol/l and ≥8.5 mmol/l glucose, respectively.

Cases with documented fetal anomalies, chromosomal abnormalities, families with history of inherited diseases and patients with known pre-existing diabetes mellitus, chronic hypertension and chronic renal disease were excluded from the REPROMETA study.

Assessment of infant growth in the REPROMETA study

Data on newborn's postnatal height and weight were collected from questionnaires filled by the mother or general practitioners at 6 and 12 months. In the genetic association testing, the postnatal age of infants born prematurely (< 259 gestational days) was corrected for their gestational age at birth. For assessment of height and weight parameters, WHO Child Growth Standards were applied (https://www.who.int/childgrowth/standards/en/).

Placental sampling of REPROMETA and HAPPY PREGNANCY study subjects

Placental sampling has been described in detail recently (Reiman et al., 2017; Sõber et al., 2015). Placental sampling was conducted within 1 h after caesarean section or vaginal delivery, placentas kept at +4C meanwhile. A full-thickness block of 2 cm was taken from the middle region of each placenta. In HAPPY PREGNANCY study, this step was repeated for each quadrant of placenta. The excision site was chosen away from the umbilical cord insertion site, large vessels and any visible or palpable infarction, hematoma, or damage. Samples were washed with 1xPBS to remove maternal blood and divided for both DNA and RNA extraction at a later date. Sections of 1 g or 100 mg of tissue for RNA extraction were placed into 10 ml or 1 ml RNAlater solution (AM7021; Thermo Fisher Scientific) in REPROMETA and HAPPY PREGNANCY study, respectively. Samples were kept at +4C for 1-3 days in RNAlater and then stored at -80C until RNA extraction. Placental DNA was extracted using NucleoSpin Tissue kit (Macherey-Nagel, Germany) according to the manufacturer's instruction.

RNA extraction and sequencing

A 200-300 mg slice of placental sample was homogenized with an IKA Ultra-Turrax T8 homogenizer (IKA Works, Staufen im Breisgau, Germany) in 4 ml Trizol reagent (15596018; Thermo Fisher Scientific). RNA was isolated using the Trizol protocol and purified with RNeasy MinElute columns (74204; Qiagen, Germantown, MD, USA) according to the manufacturer's protocol. Purity level and concentration of isolated total RNA was measured using NanoDrop® ND-1000 UV-Vis spectrophotometer (Applied Biosystems, Foster City, USA) and RIN (RNA integrity number) was estimated using Agilent 2100 Bioanalyzer (Agilent Technologies, USA).

High-purity DNA-free total RNA (5 mg) was used for rRNA depletion (Ribo-Zero rRNA Removal Kit, MRZH11124; Illumina, San Diego, CA, USA) and library preparation with Nextera Technology (FC-121-1030; Illumina). Total RNA sequencing of 40 placental samples from REPROMETA cohort was conducted in Finland Institute for Molecular Medicine (FIMM) Sequencing Core Laboratory on Illumina Hiseq2000 using 46 bp paired-end reads. Initial data refinement was performed with RNA-Seq pipeline v.2.4 (FIMM; Helsinki, Finland), reads were filtered for quality; adapter, rRNA, mitochondrial DNA sequences and homopolymer stretches. Human genome assembly (GRCh37.p7/hg19) from Ensembl v67 was used as a reference. Gene expression was quantified by HTSeq analysis (as raw read counts) and later normalized (as RPKM) using DESeq package for R. Full details of RNA extraction, library preparation, RNA-Seq and basic bioinformatics of raw data are provided elsewhere recently (Kasak et al., 2015).

Linkage disequilibrium analysis and assessment of independent eQTL signals

Linkage disequilibrium (LD) between the identified eSNPs was estimated for the REPROMETA discovery set using Haploview program (Barrett et al., 2005). eSNPs in high LD (r^2>0.8) were clustered into LD-groups and only the empirically selected lead SNP with the lowest p-value was taken forward to represent the LD-group in subsequent analyses.

Taqman RT-qPCR validation of *cis*-eQTLs in REPROMETA placental samples

Experimental validation of the discovery study targeted protein coding genes with multiple identified *cis*-SNPs that exhibited extremely low FDR and at least two-fold gene expression difference between the placentas with heterozygote and major homozygote genotypes. The *cis*-eSNPs selected for the validation were rs1150707 (*ZSCAN9* c.568+1990 C>t, reference sequence NM_001199480.1); rs10044354 (*ERAP2* g.96984791 C>t, NC_000005.10) and rs11678251 (*ALPG* c.-318 G>a, NM_031313.2). For these three SNPs, an extended REPROMETA placental sample set (n=336; **Table 2**) was genotyped using Sequenom iPLEX Gold genotyping system according to the manufacturer's protocol (Sequenom, Agena, USA). Genotyping primers are provided in **Table S1**.

Based on the genotyping outcome, 24 placentas per each eSNP were selected for the Taqman RT-qPCR gene expression quantification aiming at similar representation of alternative genotype subgroups (major and minor allele homozygotes, heterozygotes; **Table S2**). Gene expression was quantified by singleplex RT-qPCR of the target gene and housekeeping gene Ubiquitin C (UBC) as a reference gene using pre-made TaqMan Gene Expression Assays (**Table S3,** Applied Biosystems, Foster City, USA). cDNA was synthesized from 1μg total RNA according to the manufacturer's instructions (SuperScript III Reverse Transcriptase,

Applied Biosystems, Foster City, USA). All qPCR reactions were performed in triplicate in ABI 7900HT Real-time PCR system (Applied Biosystems, Foster City, USA) using HOT FIREPol® Probe qPCR Mix (Solis BioDyne, Tartu, Estonia). Relative mRNA expression values were determined by comparative CT method that accounted for mean values of normalized expression calculated by averaging three independently measured normalized expression values of the triplicate.

Association testing between SNP genotypes and estimated gene expression levels was conducted with Matrix eQTL software (Shabalin, 2012) using linear regression with additive model, adjusted for the clinical subgroups, labor activity, newborn sex and gestational age as covariates. Gestational age was added as a cofactor to account for the wider range of gestational age in validation samples. Taqman RT-qPCR experiments and respective testing of eQTL effects also targeted three additional genes (*ALPP*, *ERAP1*, *LNPEP*) that were flanking and functionally linked to the two tested primary eGenes *ALPG* and *ERAP2*. To compare results of validation to the primary analysis, the expression levels for all samples were transformed to represent the fold-change from the median expression of major homozygotes used as the reference.

Supplementary Table S1 Primer sequences for genotyping using Sequenom iPLEX Gold platform.

Primer ID	Primer sequence
rs10044354_EXT	CTTCTTCACCTTTTCAAATACT
rs10044354_FWD	ACGTTGGATGCACACTTGTGTTACCCACTC
rs10044354_REV	ACGTTGGATGCATGCAGAAAACCTGAAGAG
rs1150707_EXT	AGACGTTTCTCTATATAGTCTG
rs1150707_FWD	ACGTTGGATGCCTAGAGGTAGCTACTCTTG
rs1150707_REV	ACGTTGGATGGAAAAATATGGGAAGATAC
rs11678251_EXT	CTCCCCCAACCCCTGCT
rs11678251_FWD	ACGTTGGATGGATGAACGAGTGACAGGGTG
rs11678251_REV	ACGTTGGATGGGTATAGTGCAGTGTGCATC

Experimental validation of the discovery study targeted protein coding genes with multiple identified *cis*-eQTLs that exhibited extremely low FDR and at least two-fold gene expression difference between the placentas with heterozygote and major homozygote genotypes. The *cis*-eSNPs selected for the validation were rs1150707 (*ZSCAN9* c.568+1990 C>t, reference sequence NM_001199480.1, MAF=30%); rs10044354 (*ERAP2* g.96984791 C>t, NC_000005.10, MAF=41%) and rs11678251 (*ALPG* c.-318 G>a, NM_031313.2, MAF=11%). For these three SNPs, an extended REPROMETA placental sample set (n=336) was genotyped using Sequenom iPLEX Gold genotyping system according to the manufacturer's protocol (Sequenom, Agena, USA).

EXT, extension primers; FWD, forward primers; REV, reverse primers

Supplementary Table S2 Clinical parameters of the REPROMETA cases in the placental cis-eQTL discovery and in the validation of the *cis*-eSNP/eGene pair.

Parameter	Unit	Placental eQTL discovery analysis (n=40) Min Hom /Het /Maj Hom	Samples used for the eSNP–eGene pair validation[a]		
			***ALPG* c.-318 G>a**	***ERAP2* g.96984791 C>t**	***ZSCAN9* c.568+1990 C>t**
No of placentas per genotype	n	*ALPG*: 0/9/31<br>*ERAP2*: 8/17/15<br>*ZSCAN9*: 3/18/19	AA 4<br>GA 12<br>GG 8	TT 9<br>CT 8<br>CC 7	CC 8<br>CT 8<br>TT 8
No of placentas overlapping with the discovery analysis	n	n.a	GG 3	CC 3	CC 3<br>CT 1<br>TT 1
Clinical characteristics					
Gestational age	day	274.0 [260-284]	273.17 [232-295]	272.75 [216-293]	273.96 [216-295]
Newborn birth weight	gram	3587 [2004-4986]	3492 [1170-5182]	3560.38 [1530-5182]	3590.33 [1530-5182]
Placental weight	gram	584.0 [200-1060]	547.5 [250-860]	566.71 [250-910]	557.71 [200-910]
Newborn sex: female/male	n	21/19	11/13	12/12	14/10
Newborn length	cm	50.2 [45-55]	49.54 [37-55]	49.54 [39-55]	49.71 [39-55]
Newborn head circumference	cm	35.1 [31.5-38.5]	34.77 [29.5-39]	34.79 [30-38]	34.81 [30-38]
Newborn chest circumference	cm	34.1 [28-39]	33.42 [21-39]	33.83 [26-40.5]	34.1 [26-40.5]
Delivery: EmCS/ECS/Vag	n [%]	9/12/19<br>[22.5%/ 30.0%/47.5%]	8/3/13<br>[33.3%/12.5%/12.5 %]	6/3/15<br>[25.0%/12.5%/62.5%]	5/4/15<br>[20.8%/16.7%/62.5%]
Labor activity: no/yes/NA	n [%]	19/20/1<br>[47.5%/ 50.0%/2.5%]	6/15/3	6/17/1<br>[25%/70.8%/4.2%]	6/17/1<br>[25.0%/70.8%/4.2%]

			[25.0%/62.5%/12.5 %]		
Preeclampsia	n [%]	8 [20%]	0 [0%]	0 [0%]	1 [4.2%]
Gestational diabetes	n [%]	8 [20%]	6 [25.0%]	4 [16.7%]	5 [20.8%]
Small for gestational age	n [%]	8 [20%]	8 [33.3%]	7 [29.2%]	6 [25.0%]
Large for gestational age	n [%]	8 [20%]	7 [29.2%]	8 [33.3%]	8 [33.3%]
Maternal age	year	28.45 [18-39]	29.96 [21-40]	30.79 [21-40]	29.17 [21-40]
Maternal height	cm	166.6 [153-179]	168.08 [150-180]	168.38 [150-178]	169.06 [150-179]
Maternal pre-pregnancy weight	kg	67.79 [47-122]	67.75 [46-142]	68.46 [48-100]	68.46 [46-100]
Gestational weight gain	kg	15.56 [6-33]	14.53 [3.8-39.3]	15.59 [6-26]	17.9 [6-39.3]
Nulliparous	%	21 [52.50%]	10 [41.7%]	9 [37.5%]	12 [50%]
Maternal smoking: no/yes	n [%]	37/3 [92.5%/ 7.5%]	23/1 [95.8%/4.2%]	22/1/1 [91.7%/4.2%/4.2%]	23/1/0 [95.8%/4.2%/0%]
Paternal age	year	32.31 [21-50]	31.29 [21-40]	31.43 [19-42]	30.57 [21-42]
Paternal height	cm	183.1 [172-197.5]	178.5 [172-191]	181.05 [166-197]	180.35 [172-191]
Paternal weight	kg	91.37 [63-140]	84.15 [63-113]	87.43 [63-122]	85.73 [63-118]

Data is given as mean [minimum-maximum] unless indicated differently.

[a] Experimental validation of the discovery study targeted protein coding genes with multiple identified *cis*-eQTLs that exhibited extremely low FDR and at least two-fold gene expression difference between the placentas with heterozygote and major homozygote genotypes. The *cis*-eSNPs selected for the validation were rs1150707 (*ZSCAN9* c.568+1990 C>t, reference sequence NM_001199480.1, MAF=30%); rs10044354 (*ERAP2* g.96984791 C>t, NC_000005.10, MAF=41%) and rs11678251 (*ALPG* c.-318 G>a, NM_031313.2, MAF=11%). For these three SNPs, an extended REPROMETA placental sample set (n=336) was genotyped using Sequenom iPLEX Gold genotyping system according to the manufacturer's protocol (Sequenom, Agena, USA). Based on the genotyping outcome, 24 placentas per each eSNP were selected for the Taqman RT-qPCR gene expression quantification taking into account alike representation of alternative genotype subgroups (major and minor allele homozygotes, heterozygotes).

ALPG, Alkaline phosphatase, placental like 2; *ERAP2*, Endoplasmic reticulum aminopeptidase 2; *ZSCAN9*, Zinc finger and SCAN domain containing 9; n.a, not applicable

Supplementary Table S3 Taqman Assay probes for gene expression quantification with RT-qPCR.

Gene ID	Gene name	Assay ID
ALPP	Alkaline phosphatase, placental	Hs03046558_s1
ALPG	Alkaline phosphatase, germ cell	Hs00741068_g1
ERAP1	Endoplasmic reticulum aminopeptidase 1	Hs00429970_m1
ERAP2	Endoplasmic reticulum aminopeptidase 2	Hs01073631_m1
LNPEP	Leucyl and cystinyl aminopeptidase	Hs00893646_m1
ZSCAN9	Zinc finger and SCAN domain containing 9	Hs00196838_m1
UBC	Ubiquitin C	Hs00824723_m1

Gene expression was quantified by singleplex Taqman RT-qPCR of the target gene and housekeeping gene Ubiquitin C (UBC) as a reference gene using pre-made TaqMan Gene Expression Assays (Applied Biosystems, Foster City, USA).

Supplementary Table S4 Allele and genotype frequencies of SNPs taken forward to the genetic association testing with newborn parameters.

Gene	SNP	Samples	n	Alleles	Frequency(%)
Minor allele frequencies					
ALPG	rs11678251	**eQTL Discovery**			
		REPROMETA	40	G>A	11.3
		**Association testing**			
		REPROMETA	336		11.2
		HAPPY PREGNANCY	408		10.9
	rs744873	ALSPAC	7669	A>G	8.7
ERAP2	rs10044354	**eQTL Discovery**			
		REPROMETA	40	C>T	41.3
		**Association testing**			
		REPROMETA	350		42.0
ZSCAN9	rs1150707	**eQTL Discovery**			
		REPROMETA	40	C>T	30.0
		**Association testing**			
		REPROMETA	336		35.1
Genotype frequencies					
ALPG	rs11678251	**eQTL Discovery**	40	AA	0
		REPROMETA		GA	22.5
				GG	77.5
		**Association testing**	336	AA	1.19
		REPROMETA		GA	20.6
				GG	78.3
		HAPPY PREGNANCY	408	AA	1.64
				AG	18.0
				GG	80.4
	rs744873	ALSPAC	7669	GG	1.0
				AG	16.0
				AA	83.0
ERAP2	rs10044354	**eQTL Discovery**	40	CC	37.5
		REPROMETA		CT	42.5
				TT	20.0
		**Association testing**	336	CC	34.9
		REPROMETA		CT	46.0
				TT	19.1
ZSCAN9	rs1150707	**eQTL Discovery**	40	CC	47.5
		REPROMETA		CT	45.0
				TT	7.5
		**Association testing**	336	CC	40.9
		REPROMETA		CT	48.6
				TT	10.6

Supplementary Table S5 Growth parameters of REPROMETA children at 6 and 12 months of age.

**Age (months)**	**Sex: female/male (n)**	**Weight (g)**	**Height (cm)**	**BMI (kg/cm2)**
6	122/111	8206.2 [4510-12000]	68.5 [54-79.5]	17.4 [12.7-23.1]
12	112/104	10291.1 [5840-16700]	77.2 [58-90]	17.2 [12.1-24.5]

Data is given as mean [minimum-maximum]

Supplementary Table S7 Chromosomal localization of identified *cis*-eSNPs/eGene pairs.

**Chr**	***cis*-eSNP counts**			**eGenes**	**SNPs/Mbp**	**Genes/Mbp**
	**All**	**LD groups**	**Single**			
1	10	2	2	*PPIE, CNIH4, ATP1A4*	0.016	0.012
2	15	3	6	*THNSL2, TSGA10, ALPG, ADAM17, EPB41L5, GLS, IL36RN, THUMPD2, TTLL4*	0.037	0.037
3	1	0	1	*CBLB*	0.005	0.005
4	7	0	7	*ATP8A1, RBPJ, RPL9, SNX25*	0.037	0.021
5	23	5	3	*ATG10, ERAP2, SLC27A6, CEP72*	0.044	0.022
6	29	6	6	*ZSCAN9, PEX6, FO393415.1, IP6K3, PLEKHG1*	0.070	0.029
7	13	2	0	*ABHD11, WDR91*	0.013	0.019
8	1	0	1	*TCIM*[a]	0.007	0.007
9	7	1	4	*PSMD5, NMRK1, SLC44A1, TPRN, YBX1P6*	0.036	0.036
10	5	1	0	*IFIT5*	0.007	0.007
11	14	4	3	*TRIM5, TRIM66, PRRG4, AQP11*	0.052	0.030
12	9	2	5	*RAD52, DDX11, HTR7P1, SPSB2*	0.053	0.030
13	6	1	4	*GUCY1B2, DNAJC15, RFC3*	0.044	0.026
14	5	2	1	*HEATR5A, CEP128, HEATR4*	0.028	0.028
15	5	1	3	*NEO1*	0.040	0.010
16	14	4	3	*DCTN5, TLDC1, SMG1P5*	0.079	0.034
17	1	0	1	*SLFN5*	0.013	0.013
18	0	0	0	0	0	0
19	24	6	4	*ZNF266, PSG7, LYPD5, TMC4, ZNF749, ZNF100*	0.157	0.094
20	0	0	0	0	0	0
21	0	0	0	0	0	0
22	7	2	0	*FAM118A*	0.040	0.020
X	2	1	0	*SLC25A43*	0.006	0.006

[a] alias *c8orf4*.

Chr, chromosome; eGene, gene associated with eSNP (eQTL); *cis*-eSNP, single nucleotide variant that modulates expression of a gene or group of genes within ±100kbp from the variant; Mbp, one million basepairs; LD, linkage disequilibrium; eQTL, expression quantitative trait locus.

Supplementary Table S8 Results of eSNP validation with Taqman RT-qPCR in the REPROMETA placental samples (n=24/gene).

***Cis*-eSNP**			**Median fc(UBC) by genotype**			**Fold change[a]**			
**Chr:position**	**Alleles (MAF)**	**Gene**	**Maj hom**	**Het**	**Min hom**	**Het/Maj hom**	**Min/Maj hom**	***P*-value**	**FDR**
rs11678251 2:232406577	G>A (0.11)	*ALPP*	3.54	2.98	1.39	0.84	0.39	5.3×10^{-1}	5.3×10^{-1}
		ALPG	6.10×10^{-5}	1.5×10^{-4}	1.4×10^{-4}	2.46	2.3	1.2×10^{-1}	1.8×10^{-1}
		ALPP/ALPG	40729	19114	7119	0.47	0.17	$\mathbf{4.9\times10^{-3}}$	$\mathbf{1.2\times10^{-2}}$
rs10044354 5:96984791	C>T (0.42)	*ERAP2*	0.05	0.18	0.24	3.6	4.8	$\mathbf{3.4\times10^{-10}}$	$\mathbf{1.2\times10^{-9}}$
		ERAP1	0.05	0.05	0.06	1	1.2	1.0×10^{-1}	1.7×10^{-1}
		LNPEP	0.55	0.41	0.4	0.75	0.73	4.2×10^{-1}	4.9×10^{-1}
rs1150707 6:28229827	C>T (0.35)	*ZSCAN9*	4.23×10^{-4}	7.26×10^{-4}	1.50×10^{-3}	1.72	3.52	$\mathbf{5.1\times10^{-12}}$	$\mathbf{3.6\times10^{-11}}$

Experimental validation of the discovery study targeted protein coding genes with multiple identified *cis*-eQTLs that exhibited extremely low FDR and at least two-fold gene expression difference between the placentas with heterozygote and major homozygote genotypes. For three SNPs selected for the validation, an extended REPROMETA placental sample set (n=336) was genotyped using Sequenom iPLEX Gold genotyping system according to the manufacturer's protocol (Sequenom, Agena, USA). Based on the genotyping outcome, 24 placentas per each eSNP were selected for the Taqman RT-qPCR gene expression quantification aiming to maximize equal representation of alternative genotype subgroups (see **Supplementary Table S2**). Gene expression was quantified by singleplex Taqman RT-qPCR of the target gene and housekeeping gene Ubiquitin C (UBC) as a reference gene using pre-made TaqMan Gene Expression Assays (Applied Biosystems, Foster City, USA). Analysis was performed using linear regression with covariates (newborn birth weight, placental weight, delivery mode and gestational age). P <0.05 are marked **in bold**.
[a] fold change compared to median normalized read count of major homozygotes.
MAF, minor allele frequency; *ALPP*, Alkaline phosphatase, placental; *ALPG*, Alkaline phosphatase, placental like 2; chr, chromosome; *ERAP1*, Endoplasmic reticulum aminopeptidase 1; *ERAP2*, Endoplasmic reticulum aminopeptidase 2; fc, fold change; *HBA2(1)*, Hemoglobin subunit alpha 2(1); *LNPEP*, Leucyl and cystinyl aminopeptidase; *ZSCAN9*, Zinc finger and SCAN domain containing 9.

Supplementary Table S9 Scientific literature data for the *ALPG*, *ALPP*, *ERAP2*, *ERAP1*, *LNPEP* and *ZSCAN9* genes.

Gene ID<br>Full name	mRNA/protein expression in tissue	Implications in literature[a]	GWAS loci in the region[b]
ALPP<br>alkaline phosphatase, placental	Placenta, cervix, uterine / placenta, uterine	implantation success(Vatin et al., 2014), endometriosis(Kang et al., 1990), preterm delivery(Meyer et al., 1995), growth and remodeling of fetal tissues(She et al., 2000), preeclampsia(Chaparro et al., 2016; Orozco et al., 2009), testicular germ cell tumors(Epenetos et al., 1984)	height(Estrada et al., 2009), hip circumference(Shungin et al., 2015), waist circumference(Graff et al., 2017; Shungin et al., 2015)
ALPG<br>alkaline phosphatase, germ cell	fallopian tube, cervix, uterine / placenta	pancreatic cancer(Dua et al., 2015)	waist circumference(Graff et al., 2017; Shungin et al., 2015), schizophrenia drug response(Drago and Kure Fischer, 2018), Cognitive empathy(Warrier et al., 2018)
ERAP1[c]<br>endoplasmic reticulum aminopeptidase 1	highest in placenta / all	preeclampsia(Yong et al., 2014), Behçet's Disease(Kirino et al., 2013), essential hypertension(Yang et al., 2015), ankylosing spondylitis(Maksymowych et al., 2009), psoriasis(Sun et al., 2010), cervical carcinoma(Mehta et al., 2007), multiple sclerosis(Guerini et al., 2012)	Psoriasis(Baurecht et al., 2015; Strange et al., 2010; Stuart et al., 2015), Hodgkin's lymphoma(Urayama et al., 2012),<br>alcohol dependence(Treutlein et al., 2009), inflammatory skin disease(Baurecht et al., 2015), ankylosing spondylitis(Evans et al., 2011; Reveille et al., 2010), colorectal cancer(Al-Tassan et al., 2015)
ERAP2<br>endoplasmic reticulum aminopeptidase 2	mixed, highest in lymph node / mixed, highest skin, lung	dystocic labor(Brennan et al., 2011), preeclampsia(Hill et al., 2011; Johnson et al., 2009; Yong et al., 2014), ankylosing spondylitis(Tsui et al., 2010), psoriatic arthritis(Popa et al., 2016)	urate levels(Huffman et al., 2015), celiac disease(Li et al., 2015), juvenile idiopathic arthritis(Li et al., 2015), Crohn's disease(de Lange et al., 2017; Franke et al., 2010; Li et al., 2015; Liu et al., 2015), inflammatory bowel disease(de Lange et al., 2017; Jostins et al., 2012; Liu et al., 2015), ulcerative

			colitis(Liu et al., 2015), birdshot chorioretinopathy(Kuiper et al., 2014)
LNPEP leucyl and cystinyl aminopeptidase	mixed, highest in parathyroid gland / all	preeclampsia(Yong et al., 2014), preterm birth(Kim et al., 2013), psoriasis(Cheng et al., 2014)	urate levels(Huffman et al., 2015), celiac disease(Li et al., 2015), juvenile idiopathic arthritis(Li et al., 2015), Crohn's disease(de Lange et al., 2017; Franke et al., 2010; Li et al., 2015; Liu et al., 2015), inflammatory bowel disease(de Lange et al., 2017; Jostins et al., 2012; Liu et al., 2015), ulcerative colitis(Liu et al., 2015), birdshot chorioretinopathy(Kuiper et al., 2014)
ZSCAN9	All, highest in testis / Mixed	X-chromosome inactivation(Luijk et al., 2018)	shingles(Tian et al., 2017), urinary tract infection frequency(Tian et al., 2017), schizophrenia(Yue et al., 2011), squamous cell lung carcinoma(McKay et al., 2017), myopia(Pickrell et al., 2016), lung cancer(McKay et al., 2017), depression(Okbay et al., 2016)

[a] data from the Human Protein Atlas (https://www.proteinatlas.org/)

[b] located ±100kbp from the gene

[c] the majority of GWAS hits associated with *ERAP1* gene are also located < 100 kbp form the *ERAP2* gene

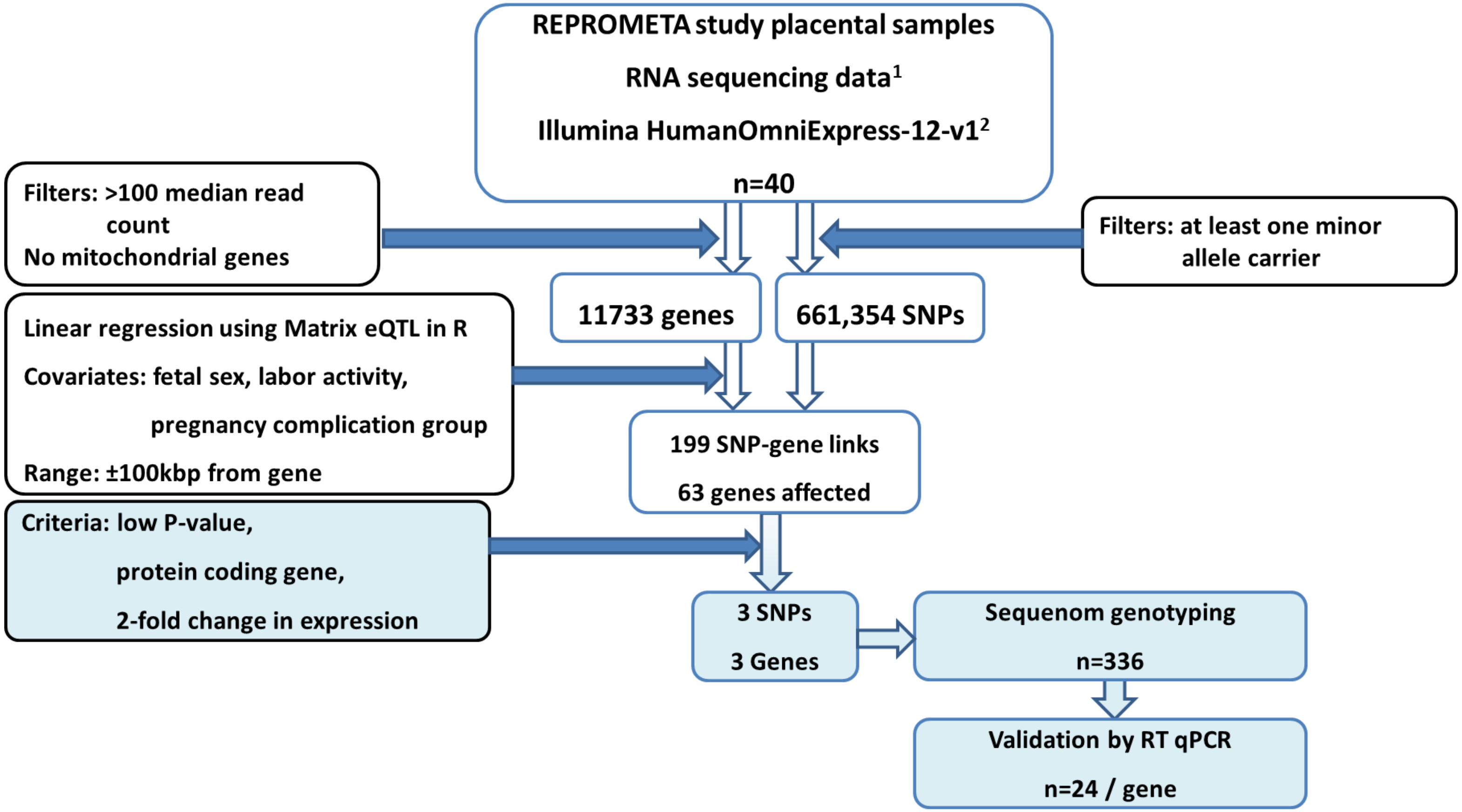

Supplementary Figure S1. Workflow of the eQTL identification and validation.
Data relevant to the discovery analysis is provided in white boxes and information on the validation experiments using Taqman RT-qPCR and Sequenom iPLEX Gold genotyping is shown in blue boxes. The discovery analysis utilized previously published placental datasets by Sõber et al., 2015 (1) and Kasak et al., 2015 (2). In linear regression analysis of genome-wide *cis*-eQTLs, statistical significance level was set to FDR< 0.05. RT-qPCR, reverse transcription-quantitative PCR.

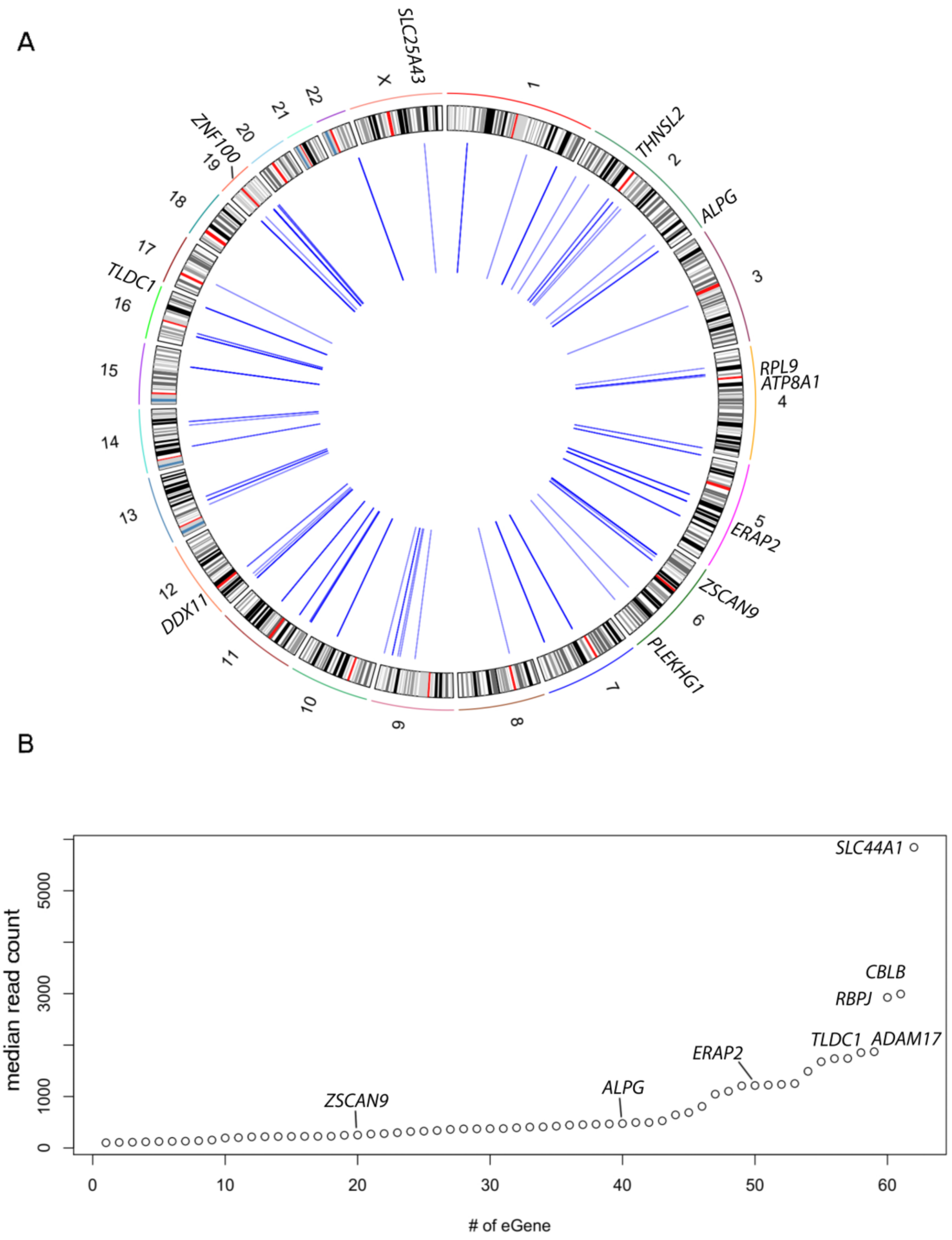

Supplementary Figure S2. Chromosomal localization and gene expression level of eGenes. **(A)** Chromosomal distribution of identified *cis*-eSNP/eGene pairs, shown by blue lines and 10 most significant *cis*-eSNP/eGene associations are indicated by gene name. Numbers 1-22 and letter X refer to chromosomes 1-22 and X, respectively. Red lines on the chromosome ideograms mark the location of centromeres. Figure was prepared with RCircos package for R. **(B)** eGenes ordered by the level of gene expression. Five most highly expressed eGenes and the three eGenes selected for validation (*ERAP2*, *ALPG and ZSCAN9*) are highlighted.

References to Supplementary Material

Al-Tassan, N. A., Whiffin, N., Hosking, F. J., Palles, C., Farrington, S. M., Dobbins, S. E., et al. (2015). A new GWAS and meta-analysis with 1000Genomes imputation identifies novel risk variants for colorectal cancer. *Sci. Rep.* 5, 10442. doi:10.1038/srep10442.

Barrett, J. C., Fry, B., Maller, J., and Daly, M. J. (2005). Haploview: analysis and visualization of LD and haplotype maps. *Bioinformatics* 21, 263–265. doi:10.1093/bioinformatics/bth457.

Baurecht, H., Hotze, M., Brand, S., Büning, C., Cormican, P., Corvin, A., et al. (2015). Genome-wide Comparative Analysis of Atopic Dermatitis and Psoriasis Gives Insight into Opposing Genetic Mechanisms. *Am. J. Hum. Genet.* 96, 104–120. doi:10.1016/j.ajhg.2014.12.004.

Brennan, D. J., McGee, S. F., Rexhepaj, E., O'Connor, D. P., Robson, M., and O'Herlihy, C. (2011). Identification of a myometrial molecular profile for dystocic labor. *BMC Pregnancy Childbirth* 11, 74. doi:10.1186/1471-2393-11-74.

Brown, M. A., Magee, L. A., Kenny, L. C., Karumanchi, S. A., McCarthy, F. P., Saito, S., et al. (2018). Hypertensive Disorders of Pregnancy. *Hypertension* 72, 24–43. doi:10.1161/HYPERTENSIONAHA.117.10803.

Chaparro, A., Gaedechens, D., Ramírez, V., Zuñiga, E., Kusanovic, J. P., Inostroza, C., et al. (2016). Placental biomarkers and angiogenic factors in oral fluids of patients with preeclampsia. *Prenat. Diagn.* 36, 476–82. doi:10.1002/pd.4811.

Cheng, H., Li, Y., Zuo, X.-B., Tang, H.-Y., Tang, X.-F., Gao, J.-P., et al. (2014). Identification of a missense variant in LNPEP that confers psoriasis risk. *J. Invest. Dermatol.* 134, 359–65. doi:10.1038/jid.2013.317.

de Lange, K. M., Moutsianas, L., Lee, J. C., Lamb, C. A., Luo, Y., Kennedy, N. A., et al. (2017). Genome-wide association study implicates immune activation of multiple integrin genes in inflammatory bowel disease. *Nat. Genet.* 49, 256–261. doi:10.1038/ng.3760.

Drago, A., and Kure Fischer, E. (2018). A molecular pathway analysis informs the genetic risk for arrhythmias during antipsychotic treatment. *Int. Clin. Psychopharmacol.* 33, 1–14. doi:10.1097/YIC.0000000000000198.

Dua, P., S, S., Kim, S., and Lee, D. (2015). ALPPL2 Aptamer-Mediated Targeted Delivery of 5-Fluoro-2'-Deoxyuridine to Pancreatic Cancer. *Nucleic Acid Ther.* 25, 180–7. doi:10.1089/nat.2014.0516.

Epenetos, A. A., Travers, P., Gatter, K. C., Oliver, R. D., Mason, D. Y., and Bodmer, W. F. (1984). An immunohistological study of testicular germ cell tumours using two different monoclonal antibodies against placental alkaline phosphatase. *Br. J. Cancer* 49, 11–5. Available at: http://www.ncbi.nlm.nih.gov/pubmed/6362705 [Accessed July 22, 2017].

Estrada, K., Krawczak, M., Schreiber, S., van Duijn, K., Stolk, L., van Meurs, J. B. J., et al. (2009). A genome-wide association study of northwestern Europeans involves the C-type natriuretic peptide signaling pathway in the etiology of human height variation. *Hum. Mol. Genet.* 18, 3516–3524. doi:10.1093/hmg/ddp296.

Evans, D. M., Spencer, C. C. A., Pointon, J. J., Su, Z., Harvey, D., Kochan, G., et al. (2011). Interaction between ERAP1 and HLA-B27 in ankylosing spondylitis implicates peptide handling in the mechanism for HLA-B27 in disease susceptibility. *Nat. Genet.* 43, 761–767. doi:10.1038/ng.873.

Franke, A., McGovern, D. P. B., Barrett, J. C., Wang, K., Radford-Smith, G. L., Ahmad, T., et al. (2010). Genome-wide meta-analysis increases to 71 the number of confirmed Crohn's disease susceptibility loci. *Nat. Genet.* 42, 1118–1125. doi:10.1038/ng.717.

Graff, M., Scott, R. A., Justice, A. E., Young, K. L., Feitosa, M. F., Barata, L., et al. (2017). Genome-wide physical activity interactions in adiposity — A meta-analysis of 200,452

adults. *PLOS Genet.* 13, e1006528. doi:10.1371/journal.pgen.1006528.
Guerini, F. R., Cagliani, R., Forni, D., Agliardi, C., Caputo, D., Cassinotti, A., et al. (2012). A Functional Variant in ERAP1 Predisposes to Multiple Sclerosis. *PLoS One* 7, e29931. doi:10.1371/journal.pone.0029931.
Hill, L. D., Hilliard, D. D., York, T. P., Srinivas, S., Kusanovic, J. P., Gomez, R., et al. (2011). Fetal ERAP2 variation is associated with preeclampsia in African Americans in a case-control study. *BMC Med. Genet.* 12, 64. doi:10.1186/1471-2350-12-64.
Huffman, J. E., Albrecht, E., Teumer, A., Mangino, M., Kapur, K., Johnson, T., et al. (2015). Modulation of Genetic Associations with Serum Urate Levels by Body-Mass-Index in Humans. *PLoS One* 10, e0119752. doi:10.1371/journal.pone.0119752.
Johnson, M. P., Roten, L. T., Dyer, T. D., East, C. E., Forsmo, S., Blangero, J., et al. (2009). The ERAP2 gene is associated with preeclampsia in Australian and Norwegian populations. *Hum. Genet.* 126, 655–66. doi:10.1007/s00439-009-0714-x.
Jostins, L., Ripke, S., Weersma, R. K., Duerr, R. H., McGovern, D. P., Hui, K. Y., et al. (2012). Host–microbe interactions have shaped the genetic architecture of inflammatory bowel disease. *Nature* 491, 119–124. doi:10.1038/nature11582.
Kang, J. O., Hudak, W. A., Crowley, W. J., and Criswell, B. S. (1990). Placental-type alkaline phosphatase in peritoneal fluid of women with endometriosis. *Clin. Chim. Acta.* 186, 285–94. Available at: http://www.ncbi.nlm.nih.gov/pubmed/2311256 [Accessed March 2, 2016].
Kasak, L., Rull, K., Vaas, P., Teesalu, P., and Laan, M. (2015). Extensive load of somatic CNVs in the human placenta. *Sci. Rep.* 5, 8342. doi:10.1038/srep08342.
Kim, J., Stirling, K. J., Cooper, M. E., Ascoli, M., Momany, A. M., McDonald, E. L., et al. (2013). Sequence variants in oxytocin pathway genes and preterm birth: a candidate gene association study. *BMC Med. Genet.* 14, 77. doi:10.1186/1471-2350-14-77.
Kirino, Y., Bertsias, G., Ishigatsubo, Y., Mizuki, N., Tugal-Tutkun, I., Seyahi, E., et al. (2013). Genome-wide association analysis identifies new susceptibility loci for Behçet's disease and epistasis between HLA-B*51 and ERAP1. *Nat. Genet.* 45, 202–207. doi:10.1038/ng.2520.
Kuiper, J. J. W., Van Setten, J., Ripke, S., Van 'T Slot, R., Mulder, F., Missotten, T., et al. (2014). A genome-wide association study identifies a functional ERAP2 haplotype associated with birdshot chorioretinopathy. *Hum. Mol. Genet.* 23, 6081–7. doi:10.1093/hmg/ddu307.
Li, Y. R., Li, J., Zhao, S. D., Bradfield, J. P., Mentch, F. D., Maggadottir, S. M., et al. (2015). Meta-analysis of shared genetic architecture across ten pediatric autoimmune diseases. *Nat. Med.* 21, 1018–1027. doi:10.1038/nm.3933.
Liu, J. Z., van Sommeren, S., Huang, H., Ng, S. C., Alberts, R., Takahashi, A., et al. (2015). Association analyses identify 38 susceptibility loci for inflammatory bowel disease and highlight shared genetic risk across populations. *Nat. Genet.* 47, 979–986. doi:10.1038/ng.3359.
Luijk, R., Wu, H., Ward-Caviness, C. K., Hannon, E., Carnero-Montoro, E., Min, J. L., et al. (2018). Autosomal genetic variation is associated with DNA methylation in regions variably escaping X-chromosome inactivation. *Nat. Commun.* 9, 3738. doi:10.1038/s41467-018-05714-3.
Maksymowych, W. P., Inman, R. D., Gladman, D. D., Reeve, J. P., Pope, A., and Rahman, P. (2009). Association of a specific ERAP1/ARTS1 haplotype with disease susceptibility in ankylosing spondylitis. *Arthritis Rheum.* 60, 1317–1323. doi:10.1002/art.24467.
McKay, J. D., Hung, R. J., Han, Y., Zong, X., Carreras-Torres, R., Christiani, D. C., et al. (2017). Large-scale association analysis identifies new lung cancer susceptibility loci and heterogeneity in genetic susceptibility across histological subtypes. *Nat. Genet.* 49, 1126–

1132. doi:10.1038/ng.3892.
Mehta, A. M., Jordanova, E. S., van Wezel, T., Uh, H.-W., Corver, W. E., Kwappenberg, K. M. C., et al. (2007). Genetic variation of antigen processing machinery components and association with cervical carcinoma. *Genes, Chromosom. Cancer* 46, 577–586. doi:10.1002/gcc.20441.
Meyer, R. E., Thompson, S. J., Addy, C. L., Garrison, C. Z., and Best, R. G. (1995). Maternal serum placental alkaline phosphatase level and risk for preterm delivery. *Am. J. Obstet. Gynecol.* 173, 181–6. Available at: http://www.ncbi.nlm.nih.gov/pubmed/7631677 [Accessed March 2, 2016].
Okbay, A., Baselmans, B. M. L., De Neve, J.-E., Turley, P., Nivard, M. G., Fontana, M. A., et al. (2016). Genetic variants associated with subjective well-being, depressive symptoms and neuroticism identified through genome-wide analyses. *Nat. Genet.* 48, 624–633. doi:10.1038/ng.3552.
Orozco, A. F., Jorgez, C. J., Ramos-Perez, W. D., Popek, E. J., Yu, X., Kozinetz, C. A., et al. (2009). Placental release of distinct DNA-associated micro-particles into maternal circulation: reflective of gestation time and preeclampsia. *Placenta* 30, 891–7. doi:10.1016/j.placenta.2009.06.012.
Pickrell, J. K., Berisa, T., Liu, J. Z., Ségurel, L., Tung, J. Y., and Hinds, D. A. (2016). Detection and interpretation of shared genetic influences on 42 human traits. *Nat. Genet.* 48, 709–717. doi:10.1038/ng.3570.
Popa, O. M., Cherciu, M., Cherciu, L. I., Dutescu, M. I., Bojinca, M., Bojinca, V., et al. (2016). ERAP1 and ERAP2 Gene Variations Influence the Risk of Psoriatic Arthritis in Romanian Population. *Arch. Immunol. Ther. Exp. (Warsz).* 64, 123–129. doi:10.1007/s00005-016-0444-4.
Reiman, M., Laan, M., Rull, K., and Sõber, S. (2017). Effects of RNA integrity on transcript quantification by total RNA sequencing of clinically collected human placental samples. *FASEB J.* 31, 3298–3308. doi:10.1096/fj.201601031RR.
Reveille, J. D., Sims, A.-M., Danoy, P., Evans, D. M., Leo, P., Pointon, J. J., et al. (2010). Genome-wide association study of ankylosing spondylitis identifies non-MHC susceptibility loci. *Nat. Genet.* 42, 123–127. doi:10.1038/ng.513.
Shabalin, A. A. (2012). Matrix eQTL: ultra fast eQTL analysis via large matrix operations. *Bioinformatics* 28, 1353–8. doi:10.1093/bioinformatics/bts163.
She, Q. B., Mukherjee, J. J., Chung, T., and Kiss, Z. (2000). Placental alkaline phosphatase, insulin, and adenine nucleotides or adenosine synergistically promote long-term survival of serum-starved mouse embryo and human fetus fibroblasts. *Cell. Signal.* 12, 659–65. Available at: http://www.ncbi.nlm.nih.gov/pubmed/11080618 [Accessed March 2, 2016].
Shungin, D., Winkler, T. W., Croteau-Chonka, D. C., Ferreira, T., Locke, A. E., Mägi, R., et al. (2015). New genetic loci link adipose and insulin biology to body fat distribution. *Nature* 518, 187–196. doi:10.1038/nature14132.
Sildver, K., Veerus, P., and Lang, K. (2015). Sünnikaalukõverad Eestis ja sünnikaalu mõjutavad tegurid: registripõhine uuring - Eesti Arst - Eesti Arstide Liidu ajakiri. *Eesti Arst* 94, 465–470. Available at: http://eestiarst.ee/sunnikaalukoverad-eestis-ja-sunnikaalu-mojutavad-tegurid-registripohine-uuring/ [Accessed October 30, 2015].
Sõber, S., Reiman, M., Kikas, T., Rull, K., Inno, R., Vaas, P., et al. (2015). Extensive shift in placental transcriptome profile in preeclampsia and placental origin of adverse pregnancy outcomes. *Sci. Rep.* 5, 13336. doi:10.1038/srep13336.
Strange, A., Capon, F., Spencer, C. C. A., Knight, J., Weale, M. E., Allen, M. H., et al. (2010). A genome-wide association study identifies new psoriasis susceptibility loci and an interaction between HLA-C and ERAP1. *Nat. Genet.* 42, 985–990. doi:10.1038/ng.694.
Stuart, P. E., Nair, R. P., Tsoi, L. C., Tejasvi, T., Das, S., Kang, H. M., et al. (2015). Genome-

wide Association Analysis of Psoriatic Arthritis and Cutaneous Psoriasis Reveals Differences in Their Genetic Architecture. *Am. J. Hum. Genet.* 97, 816–836. doi:10.1016/j.ajhg.2015.10.019.

Sun, L.-D., Cheng, H., Wang, Z.-X., Zhang, A.-P., Wang, P.-G., Xu, J.-H., et al. (2010). Association analyses identify six new psoriasis susceptibility loci in the Chinese population. *Nat. Genet.* 42, 1005–1009. doi:10.1038/ng.690.

Tian, C., Hromatka, B. S., Kiefer, A. K., Eriksson, N., Noble, S. M., Tung, J. Y., et al. (2017). Genome-wide association and HLA region fine-mapping studies identify susceptibility loci for multiple common infections. *Nat. Commun.* 8, 599. doi:10.1038/s41467-017-00257-5.

Treutlein, J., Cichon, S., Ridinger, M., Wodarz, N., Soyka, M., Zill, P., et al. (2009). Genome-wide Association Study of Alcohol Dependence. *Arch. Gen. Psychiatry* 66, 773. doi:10.1001/archgenpsychiatry.2009.83.

Tsui, F. W. L., Haroon, N., Reveille, J. D., Rahman, P., Chiu, B., Tsui, H. W., et al. (2010). Association of an ERAP1 ERAP2 haplotype with familial ankylosing spondylitis. *Ann. Rheum. Dis.* 69, 733–6. doi:10.1136/ard.2008.103804.

Urayama, K. Y., Jarrett, R. F., Hjalgrim, H., Diepstra, A., Kamatani, Y., Chabrier, A., et al. (2012). Genome-Wide Association Study of Classical Hodgkin Lymphoma and Epstein–Barr Virus Status–Defined Subgroups. *JNCI J. Natl. Cancer Inst.* 104, 240–253. doi:10.1093/jnci/djr516.

Vatin, M., Bouvier, S., Bellazi, L., Montagutelli, X., Laissue, P., Ziyyat, A., et al. (2014). Polymorphisms of Human Placental Alkaline Phosphatase Are Associated with in Vitro Fertilization Success and Recurrent Pregnancy Loss. *Am. J. Pathol.* 184, 362–368. doi:10.1016/j.ajpath.2013.10.024.

Warrier, V., Grasby, K. L., Uzefovsky, F., Toro, R., Smith, P., Chakrabarti, B., et al. (2018). Genome-wide meta-analysis of cognitive empathy: heritability, and correlates with sex, neuropsychiatric conditions and cognition. *Mol. Psychiatry* 23, 1402–1409. doi:10.1038/mp.2017.122.

Yang, S., Liu, X., Gao, Y., Ding, M., Li, B., Sun, H., et al. (2015). Association of single nucleotide polymorphisms in the 3′UTR of ERAP1 gene with essential hypertension in the Northeastern Han Chinese. *Gene* 560, 211–216. doi:10.1016/j.gene.2015.02.005.

Yong, H. E. J., Murthi, P., Borg, A., Kalionis, B., Moses, E. K., Brennecke, S. P., et al. (2014). Increased decidual mRNA expression levels of candidate maternal pre-eclampsia susceptibility genes are associated with clinical severity. *Placenta* 35, 117–24. doi:10.1016/j.placenta.2013.11.008.

Yue, W.-H., Wang, H.-F., Sun, L.-D., Tang, F.-L., Liu, Z.-H., Zhang, H.-X., et al. (2011). Genome-wide association study identifies a susceptibility locus for schizophrenia in Han Chinese at 11p11.2. *Nat. Genet.* 43, 1228–1231. doi:10.1038/ng.979.
